# Transcription factors-related molecular subtypes and risk prognostic model: exploring the immunogenicity landscape and potential drug targets in hepatocellular carcinoma

**DOI:** 10.1186/s12935-023-03185-1

**Published:** 2024-01-04

**Authors:** Meixia Wang, Hanyao Guo, Bo Zhang, Yanan Shang, Sidi Zhang, Xiaoyu Liu, Pengxiu Cao, Yumei Fan, Ke Tan

**Affiliations:** Ministry of Education Key Laboratory of Molecular and Cellular Biology, Hebei Research Center of the Basic Discipline of Cell Biology, Hebei Province Key Laboratory of Animal Physiology, Biochemistry and Molecular Biology, College of Life Sciences, Hebei Normal University, Shijiazhuang, Hebei China

**Keywords:** Transcription factors (TF), Hepatocellular carcinoma, Molecular subtype, Drug sensitivity, Immune microenvironment

## Abstract

**Background:**

Hepatocellular carcinoma (HCC) is the most prevalent form of liver cancer, with a high mortality rate and poor prognosis. Mutated or dysregulated transcription factors (TFs) are significantly associated with carcinogenesis. The aim of this study was to develop a TF-related prognostic risk model to predict the prognosis and guide the treatment of HCC patients.

**Methods:**

RNA sequencing data were obtained from the TCGA database. The ICGC and GEO databases were used as validation datasets. The consensus clustering algorithm was used to classify the molecular subtypes of TFs. Kaplan‒Meier survival analysis and receiver operating characteristic (ROC) analysis were applied to evaluate the prognostic value of the model. The immunogenic landscape differences of molecular subtypes were evaluated by the TIMER and xCell algorithms. Autodock analysis was used to predict possible binding sites of trametinib to TFs. RT‒PCR was used to verify the effect of trametinib on the expression of core TFs.

**Results:**

According to the differential expression of TFs, HCC samples were divided into two clusters (C1 and C2). The survival time, signaling pathways, abundance of immune cell infiltration and responses to chemotherapy and immunotherapy were significantly different between C1 and C2. Nine TFs with potential prognostic value, including HMGB2, ESR1, HMGA1, MYBL2, TCF19, E2F1, FOXM1, CENPA and ZIC2, were identified by Cox regression analysis. HCC patients in the high-risk group had a poor prognosis compared with those in the low-risk group (*p* < 0.001). Moreover, the area under the ROC curve (AUC) values of the 1-year, 2-year and 3-year survival rates were 0.792, 0.71 and 0.695, respectively. The risk model was validated in the ICGC database. Notably, trametinib sensitivity was highly correlated with the expression of core TFs, and molecular docking predicted the possible binding sites of trametinib with these TFs. More importantly, the expression of core TFs was downregulated under trametinib treatment.

**Conclusions:**

A prognostic signature with 9 TFs performed well in predicting the survival rate and chemotherapy/immunotherapy effect of HCC patients. Trimetinib has potential application value in HCC by targeting TFs.

**Supplementary Information:**

The online version contains supplementary material available at 10.1186/s12935-023-03185-1.

## Introduction

According to recent advances in tumor genetics and genomics, it is becoming clear carcinogenesis is a complex process that involves interactions between multiple oncogenes and tumor suppressor genes [1]. Transcription factors (TFs) account for approximately one-fifth of the identified oncogenes and play a crucial role in carcinogenesis by regulating the expression of many downstream target genes [2]. TFs are a group of proteins with DNA-binding domain that binds to specific DNA sequences in promoter and/or enhancer regions and regulate the transcription of a wide variety of target genes. TFs are capable of regulating cell growth, cell division, cell proliferation, cell death and metastasis. Due to their importance, many studies have been undertaken to understand the molecular mechanisms of TFs in oncogenesis.

Previous studies have demonstrated that aberrations in the activity and expression of TFs are closely linked with multiple features of hepatocellular carcinoma (HCC). TFs are involved in multiple important molecular events in HCC progression, such as angiogenesis, metastasis and metabolic reprogramming. Thus, TFs are closely associated with multiple malignant features of HCC. For instance, some members of the Forkhead-box (FOX) transcription factor family contribute to the pathogenesis of HCC by activating or inhibiting different signaling pathways and cellular events [3]. The transcription factor AP-4 activated the Wnt/β-catenin pathway to promote tumorigenesis in HCC [4]. Additionally, YAP/TAZ and ATF4 jointly inhibit ferroptosis, leading to HCC cell resistance to sorafenib [5]. Targeting transcriptional dysregulation of tumor cells has been shown to be more effective than targeting core kinases of signaling pathways within tumor cells [6]. Although the crucial roles of a series of TFs in oncogenesis have been gradually recognized and revealed, most published literature focuses on the function of individual TF. In the process of tumor occurrence and development, a biological process is often regulated by multiple TFs, and TFs always interact with each other to promote or inhibit their functions. The in-depth understanding of TF regulation, including TF expression, activation, degradation, protein–protein interaction and its DNA-binding pattern dynamics, opens up new possibilities for TFs as potential cancer drug targets for HCC. Although scientists have explored the prognostic value and application of TF-associated signature in some tumors, it has not been thoroughly studied in HCC [7–14]. Moreover, the prognostic potential of TFs and their effects on the tumor microenvironment and chemotherapy/immunotherapy in HCC need further exploration. Recently, Yang et al. identified a 2-TF signature and constructed a prognostic model for HCC patients, but the relationship between TF-based signature and the tumor immune microenvironment remains unclear [15]. The aim of this study was to develop a TF-related prognostic risk model to predict the prognosis, uncover the effect of TF-related signature on immune cell infiltration, and guide the personalized treatment of HCC patients, including chemotherapy and immunotherapy.

In the present study, we used a comprehensive bioinformatics approach and machine learning algorithm to identify the expression patterns, prognostic value and molecular subtypes of TFs in HCC. We explored the prognostic ability of TF and its potential to guide clinical treatment using a completely different approach from previously published literature. We first employed consensus clustering analysis to classify patients into two distinct molecular subtypes (cluster 1 and cluster 2, C1 and C2). The differences in immune infiltration, signaling pathways, drug sensitivity and immunotherapy between different subtypes were analyzed. Notably, HCC patients in the C1 subgroup are more suitable for chemotherapy, while patients in the C2 subtype are more amenable to immunotherapy. According to these findings, we proposed a personalized treatment plan for HCC patients based on TFs-related molecular subtypes. We further developed a risk score model containing 9 genes for HCC patients based on the expression of TFs to explore the prognostic potential of TFs in HCC through Cox regression analysis. It is worth mentioning that our established 9-TF signature (1-year AUC = 0.792, 2-year AUC = 0.71 and 3-year AUC = 0.695) showed improved predictive capability for the survival rate of HCC patients compared to the previously identified 2-TF signature (1-year AUC = 0.73, 2-year AUC = 0.60 and 3-year AUC = 0.61) based on the TCGA database [15]. More importantly, we investigated the interaction between trametinib and core TFs by molecular docking. Our findings investigated the molecular mechanism of TFs in HCC progression and highlighted an unexplored avenue for TF-based classifiers as biomarkers of HCC. Our data also provide new insights into using TF to predict the prognosis of HCC patients and improve the chemotherapy effect and immunotherapy response for individualized treatment strategies.

## Materials and methods

### Data acquisition and processing

The bulk transcriptome data and clinical information of HCC samples and normal samples were downloaded from the TCGA database (https://portal.gdc.cancer.gov) and ICGC database (https://dcc.icgc.org/releases/current/Projects) on May 3, 2022. The TCGA-LIHC cohort includes 371 HCC samples and 50 normal samples, and the ICGC cohort includes 240 HCC samples and 202 normal samples. RNA-seq raw count data were normalized to TPM data, and the log2 method was used for standardization.

### Identification of differentially expressed TFs

The list of human TFs was downloaded from the HumanTFDB database (http://bioinfo.life.hust.edu.cn/HumanTFDB#!/). The differentially expressed genes (DEGs) between HCC tissues and normal liver tissues were identified by the R (Version 4.2.2) packages “limma” and “DESeq2”. Genes meeting |Log_2_(Fold Change)|> 1 and P < 0.05 were defined as DEGs. The R packages “ggplot2” and “pheatmap” were used to visualize the results of differential expression analysis. The package “ClusterProfiler” was used for the Kyoto Encyclopedia of Genes and Genomes (KEGG) and Gene Ontology (GO) enrichment analysis of upregulated and downregulated genes.

### Enrichment and correlation analysis of TFs

The protein–protein interaction (PPI) analysis and enrichment analysis of differentially expressed TFs were performed by the Metascape database (https://metascape.org/gp/index.html#/main/step1). The R package “pheatmap” was utilized to draw the correlation heatmap.

### Consensus cluster analysis and survival analysis of HCC samples

The R package “ConsensusClusterPlus” was used for consensus clustering analysis according to the expression of TFs. Principal component analysis (PCA) was used to verify the reliability of the clustering results. The R package “pheatmap” was used to visualize the clustering matrix. The R packages “survival” and “survminer” were used to analyze the survival rate of patients. Kaplan‒Meier (KM) analysis and log-rank test were used to compare the difference in survival rate between different subgroups.

### Analysis of the immune infiltration microenvironment in different clusters

Tumor Immune Estimation Resource (TIMER) and xCell algorithms were applied to analyze the infiltration abundance of six immune cells in C1 and C2 using the R package “immunedecov”. The R packages “ggplot2” and “pheatmap” were used to visualize immune cell abundance and TIDE scores.

### Mutation landscape and drug sensitivity analysis

Tumor mutational burden (TMB) was used to quantitatively analyze the difference in the mutation landscape between C1 and C2. The R package “maftools” was utilized to calculate TMB and generate a waterfall plot for visualization. We used ridge regression to calculate the half maximum inhibition concentration (IC50 value) to represent drug sensitivity. Drug sensitivity data were downloaded from the Genomics of Drug Sensitivity in Cancer (GDSC) database (https://www.cancerrxgene.org/). The R package “pRRophic” was used to calculate the IC50 value and predict drug sensitivity. The relationships between TFs expression, drug sensitivity and cancer-related signaling pathways were analyzed using the GSCALite online website (http://bioinfo.life.hust.edu.cn/web/GSCALite/).

### Cox regression analysis and risk scoring model

The R package “survival” was used for univariate and multivariate Cox regression analyses. The package “forestplot” was applied to draw forest plots and visualize the results of Cox regression analysis. The R Package “ggrisk” was used to calculate the risk score and visualize it. The risk score is calculated based on a multivariate Cox regression analysis:

$$Risk\,score\, = \,\sum \,\left( {{\text{Coefficient}}\,i\, \times \,{\text{Exp}}\,i} \right)$$

The *i* here represents the selected gene. Exp *i* represents the expression level of the prognosis-related TF. The raw data used to calculate the risk score is shown in Additional file 9: Table S1 and Additional file 10: Table S2. KM analysis and log-rank tests were used to analyze the difference in survival rate between the two groups. The R package “timeROC” was used to draw receiver operating characteristic (ROC) curves to evaluate the accuracy of the risk scoring model.

### Construction of a predictive nomogram

The “rms” package was applied to establish a nomogram based on multivariate analysis. The 1-year, 2-year and 3-year survival probabilities of HCC patients were predicted by the nomogram. The R package “survivals” was used to calculate the ROC and concordance index (C-index) to evaluate the accuracy of a nomogram as a predictor and generate calibration curves.

### Verification of the expression of nine key TFs

First, differentially expressed 40 TFs in HCC were screened using |Log2(Fold Change)|> 1 and *p* < 0.05 as the thresholds. Then, we performed a univariate Cox regression analysis based on the expression levels of identified TFs and used *p* < 0.01 as the threshold to select TFs with prognostic value. To eliminate data heterogeneity in the TCGA and ICGA databases, we then intersected the core genes screened from the two different databases and finally obtained 9 TFs for further analysis. The TNMplot database (https://tnmplot.com/analysis/) was used to analyze the expression of the TF-related signature in normal samples, HCC and metastatic samples. The GSE64041 and GSE54236 datasets were used to verify the expression of nine key TFs in HCC and normal samples. Immunohistochemical (IHC) results were obtained from the HPA database (https://www.proteinatlas.org/). Single-cell RNA-seq results were obtained from the Human Life Browser database (https://itzkovitzwebapps.weizmann.ac.il/webapps/home/session.html?app=HumanLiverBrowser).

### Molecular docking and visualization

To predict possible binding sites of 9 TFs with trametinib, we performed molecular docking analysis. The three-dimensional conformation of trametinib was downloaded from the PubChem database (https://pubchem.ncbi.nlm.nih.gov/). Protein structure data were downloaded from the PDB database (https://www.rcsb.org/) and the AlphaFold database (https://alphafold.com/). AutoDock software (version 1.5.6) was used to realize molecular docking. A genetic algorithm was used to search molecular docking conformations. OpenBabel software (version 2.4.1) was used for format conversion of docking results. PyMOL software (version 2.2.0) was used for visualization of the docking results.

### Real-time polymerase chain reaction (RT-PCR) analysis

HepG2 and Huh7 cells were purchased from the American Type Culture Collection (ATCC) and cultured in Dulbecco's modified Eagle’s medium (DMEM) supplemented with 10% fetal bovine serum (FBS) and 1% penicillin/streptomycin [16, 17]. The culture conditions were a humidified atmosphere (37 °C, 5% CO_2_). HepG2 and Huh7 cells were treated with different concentrations of trametinib. Cell viability was determined by cell counting kit-8 (CCK8) (MedChemExpress, China) [16, 17]. The expression of core TFs under trametinib treatment was detected by RT-PCR. An RNA easy kit (Vazyme, Nanjing, China) was used to extract total RNA. A reverse transcription kit and SYBR qPCR master mix (Biosharp, Beijing, China) was used for reverse transcription and RT-PCR. The primer sequences are shown in Table 1. S18 was used as the internal reference gene. Relative mRNA levels were calculated using the 2^−ΔΔCT^ method.

**Table 1:** Primers used for RT-PCR

Gene	Forward primers	Reverse primers
CENPA	GGCGGAGACAAGGTTGGCTAAA	GGCTTGCCAATTGAAGTCCACAC
E2F1	GGACCTGGAAACTGACCATCAG	CAGTGAGGTCTCATAGCGTGAC
FOXM1	TCTGCCAATGGCAAGGTCTCCT	CTGGATTCGGTCGTTTCTGCTG
HMGA1	GAAGTGCCAACACCTAAGAGACC	GGTTTCCTTCCTGGAGTTGTGG
TCF19	TCAGCCTGGAAGACCACAGCAG	CCAAAGGTCAGGAGGTCTCCAT
HMGB2	GGTGAAATGTGGTCTGAGCAGTC	CCTGCTTCACTTTTGCCCTTGG
MYBL2	CACCAGAAACGAGCCTGCCTTA	CTCAGGTCACACCAAGCATCAG
ZIC2	ACACAGGCGAGAAACCCTTCCC	ACTCACACTGGAACGGCTTCTC
S18	GTTCCGACCATAAACGATGCC	TGGTGGTGCCCTTCCGTCAAT

### Statistical method

Statistical analyses were completed by R software (version 4.2.2). The Wilcoxon test was used to compare the differences between the two groups. The log-rank test was used for the difference in KM analysis. Spearman correlation analysis was used to analyze the correlation coefficient between the two groups. *p* < 0.05 was defined as statistically significant.

## Results

### Identification of differentially expressed genes (DEGs) between HCC and normal samples

**Fig. 1:**
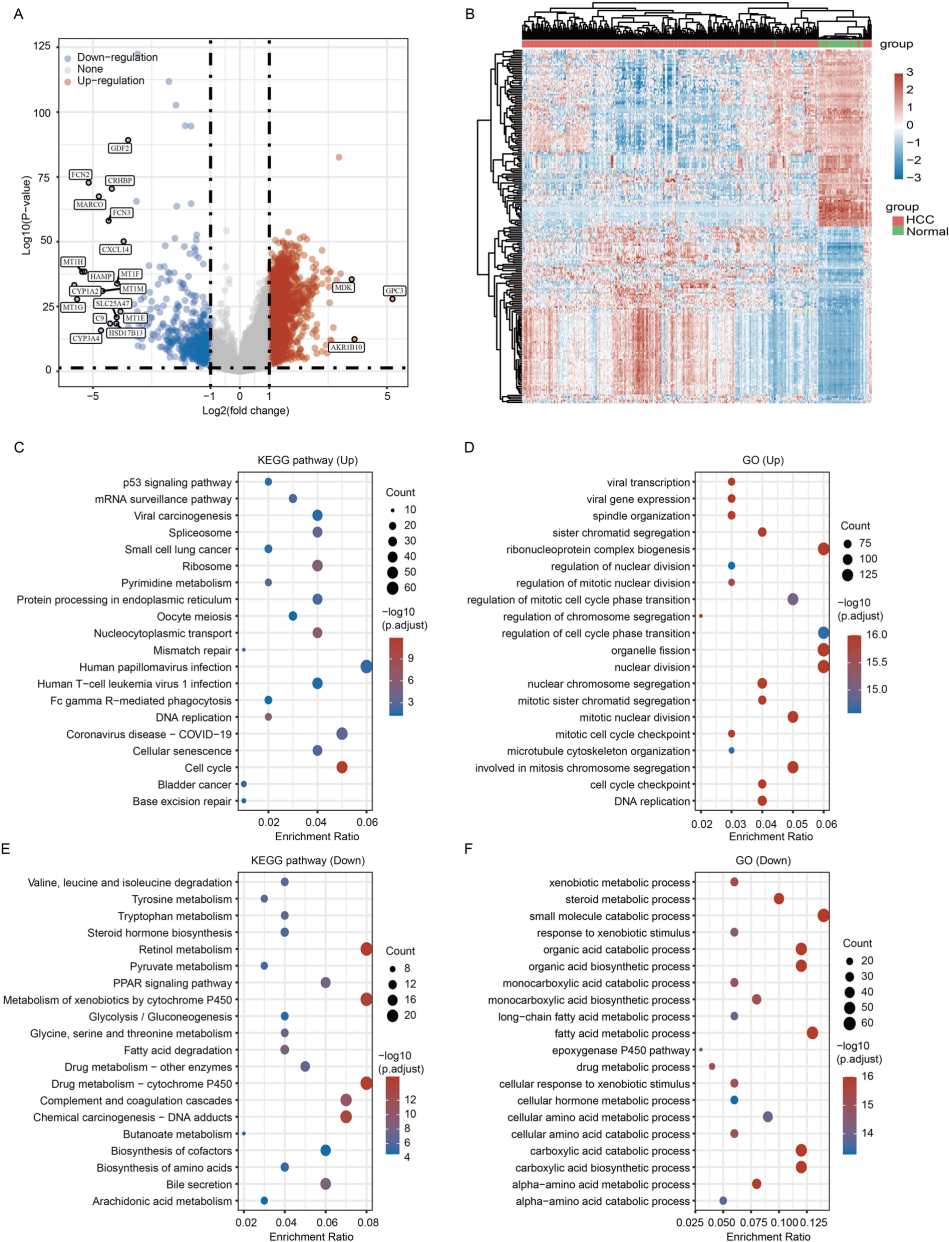
Analysis of DEGs in the TCGA database. **A** Volcano plot of DEGs between HCC tissues and normal tissues. |Log_2_(Fold Change)|> 1 and *p* < 0.05. **B** Clustering heatmap of the expression of DEGs in HCC samples and normal samples. **C** KEGG pathway analysis based on upregulated DEGs. **D** GO enrichment analysis of upregulated DEGs. **E** KEGG analysis based on downregulated DEGs. **F** GO enrichment analysis of downregulated DEGs

To identify the DEGs between HCC and normal samples, we performed differential expression analysis using the TCGA database. A total of 2897 DEGs (2451 upregulated DEGs and 446 downregulated DEGs) were obtained with |log_2_(Fold Change)|> 1 and *p* < 0.05 as the thresholds (Fig. 1A). A cluster heatmap showed the expression of these genes in HCC samples and normal samples (Fig. 1B). KEGG and GO enrichment analyses using the upregulated or downregulated genes were performed to explore the molecular mechanisms of HCC development. KEGG analysis of upregulated genes demonstrated that these genes were mainly involved in human papillomavirus infection, coronavirus disease-COVID-19, and cell cycle (Fig. 1C). GO enrichment analysis indicated that the upregulated genes were significantly associated with cell proliferation, ribonucleoprotein complex biogenesis, and regulation of cell cycle phase transition (Fig. 1D). Moreover, the KEGG pathways of downregulated genes were mainly concentrated in the metabolic process, retinol metabolism, and metabolism of xenobiotics by cytochrome P450 (Fig. 1E). The GO enrichment results of downregulated genes revealed that these genes were mainly involved in the metabolism of small molecules. The top three enrichment results were small molecule catabolic process, fatty acid metabolic process, and organic acid catabolic process (Fig. 1F). Similar analyses were also performed using the ICGC database. Based on the same criterion (|log_2_(Fold Change)|> 1 and *p* < 0.05), we identified 966 DEGs between HCC and normal liver tissues in the ICGC database, with 544 upregulated genes and 422 downregulated genes (Additional file 1: Fig. S1A, B).

### Acquisition and enrichment analysis of the TFs gene set

**Fig. 2:**
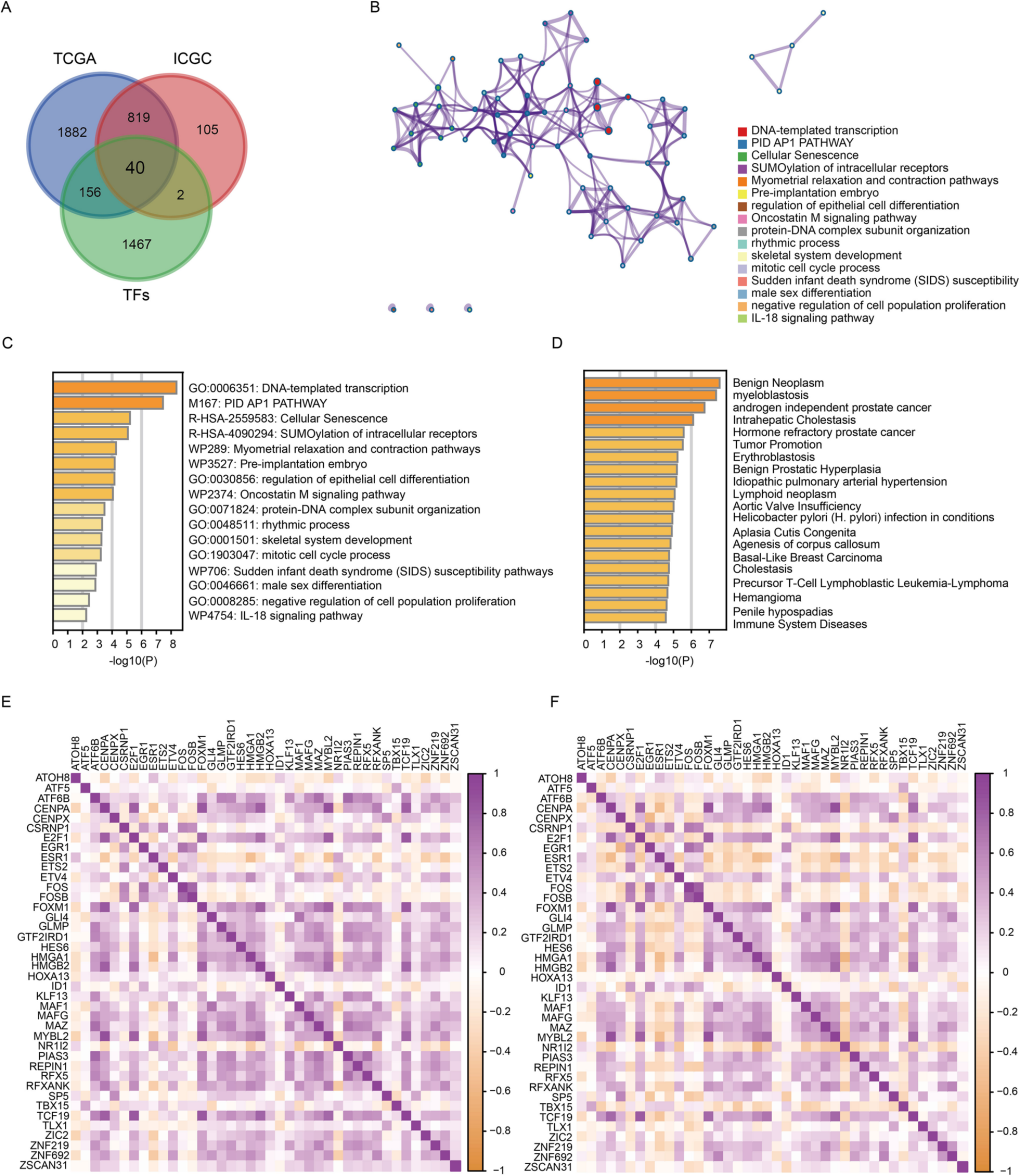
Acquisition of differentially expressed TF gene set and enrichment analysis. **A** Venn diagram showing the acquisition of the TF gene set. **B** PPI network diagram of TFs in the Metascape database. **C** Bar plots demonstrating the top 20 signaling pathways associated with 40 TFs in the Metascape database. **D** Disease enrichment analysis of TFs in the Metascape database. **E**, **F** Correlation heatmap of TF expression in the TCGA and ICGC databases

To accurately identify TFs that are differentially expressed in HCC, we downloaded a list containing 1665 human TFs from the HumanTFDB database. We intersected the differentially expressed TFs obtained from the TCGA database and ICGC database with the TFs list, and 40 genes were obtained as the TF gene set (Fig. 2A).

To deeply understand the biological function of the TF gene set, we conducted enrichment analysis and constructed a protein–protein interaction (PPI) network using these 40 genes through the Metascape database (Fig. 2B). The top three enriched signaling pathways were DNA-templated transcription, PID AP1 pathway and cellular senescence (Fig. 2C). The disease enrichment results indicated that these TFs were closely related to a variety of cancers, including androgen-independent prostate cancer, basal-like breast carcinoma, hemangioma and precursor T-cell lymphoblastic leukemia-lymphoma (Fig. 2D). In addition, some immune-related diseases were observed, such as immune system diseases (Fig. 2D). The expression of these 40 genes also exhibited good correlation in both TCGA and ICGA databases (Fig. 2E, F).

### Consensus cluster analysis of TF-related molecular subtypes in HCC patients

**Fig. 3:**
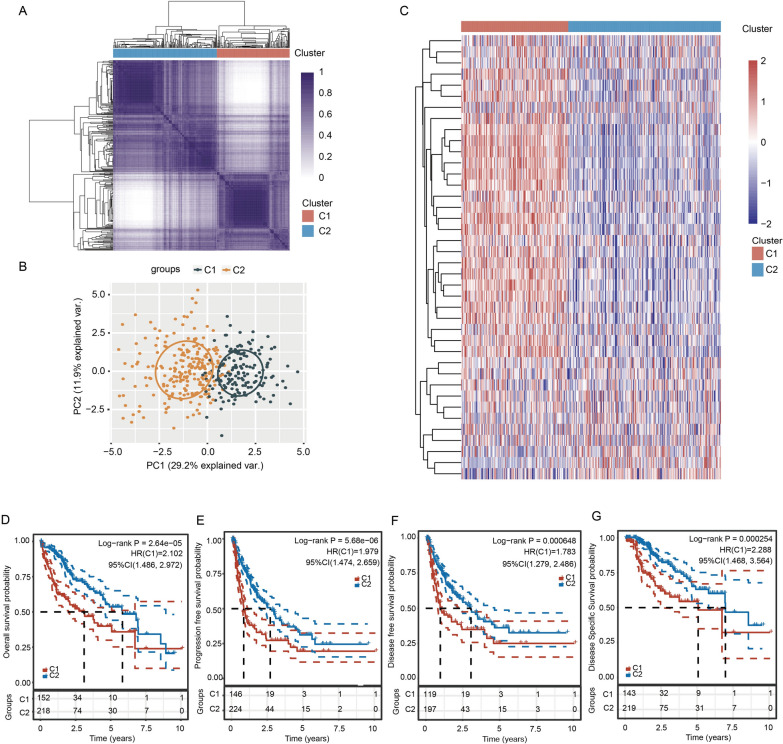
Consensus cluster analysis of TFs. **A** Consensus clustering matrix when k = 2. **B** PCA of two clusters of HCC patients. **C** Heatmap showing the expression of 40 TFs in C1 and C2. **D**–**G** KM survival curves show the difference in survival probability between C1 and C2 with the log-rank test

We then conducted consensus cluster analysis on HCC samples according to the expression of 40 TFs in the TCGA database. HCC samples were divided into two clusters with k = 2 (Fig. 3A). We further performed PCA to verify the clustering effect (Fig. 3B). The expression of most TFs in cluster 1 (C1) patients was higher than cluster 2 (C2) patients (Fig. 3C and Additional file 1: Fig. S1C). Only three TFs, including ATOH8, ESR1 and NR1I2, were lower expressed in C1 compared with C2 (Fig. 3C and Additional file 1: Fig. S1C). To explore whether there is any difference in prognosis between C1 and C2 patients, we used the KM method to analyze the difference in overall survival (OS, *p* = 2.64e-05), progression-free survival (PFS, *p* = 5.68e-06), disease-free survival (DFS, *p* = 0.000648) and disease-specific survival (DSS, *p* = 0.000254) between C1 and C2. KM analysis showed that C2 patients had better survival rates than C1 patients (Fig. 3D–G). Additionally, there were significant differences in survival status, age, T stage, TNM stage and grade between C1 and C2 (Table 2).

**Table 2:** Relationships between various clinicopathological parameters and two clusters in HCC

	Characteristic	C1	C2	*p* value
Status	Alive	87	154	
	Dead	66	64	0.009
Age	Mean (SD)	57.7 (13.3)	60.6 (13.6)	
	Median [MIN, MAX]	59 [18, 85]	63 [16, 90]	0.042
Sex	FEMALE	56	65	
	MALE	97	153	0.208
Race	AMERICAN INDIAN	1	1	
	ASIAN	73	85	
	BLACK	9	8	
	WHITE	70	114	0.364
pT-stage	T1	57	124	
	T2	46	46	
	T2a	1		
	T2b	1		
	T3	24	21	
	T3a	16	13	
	T3b	3	3	
	T4	5	8	
	TX		1	0.009
pN-stage	N0	107	145	
	N1	3	1	
	NX	42	72	0.229
pM-stage	M0	112	154	
	M1	1	3	
	MX	40	61	0.73
pTNM-stage	I	55	116	
	II	41	45	
	III	1	2	
	IIIA	36	29	
	IIIB	4	4	
	IIIC	6	3	
	IV	1	1	
	IVA		1	
	IVB		2	0.018
Grade	G1	11	44	
	G2	60	117	
	G3	73	49	
	G4	6	6	0

### Analysis of the immune infiltration microenvironment of C1 and C2

**Fig. 4:**
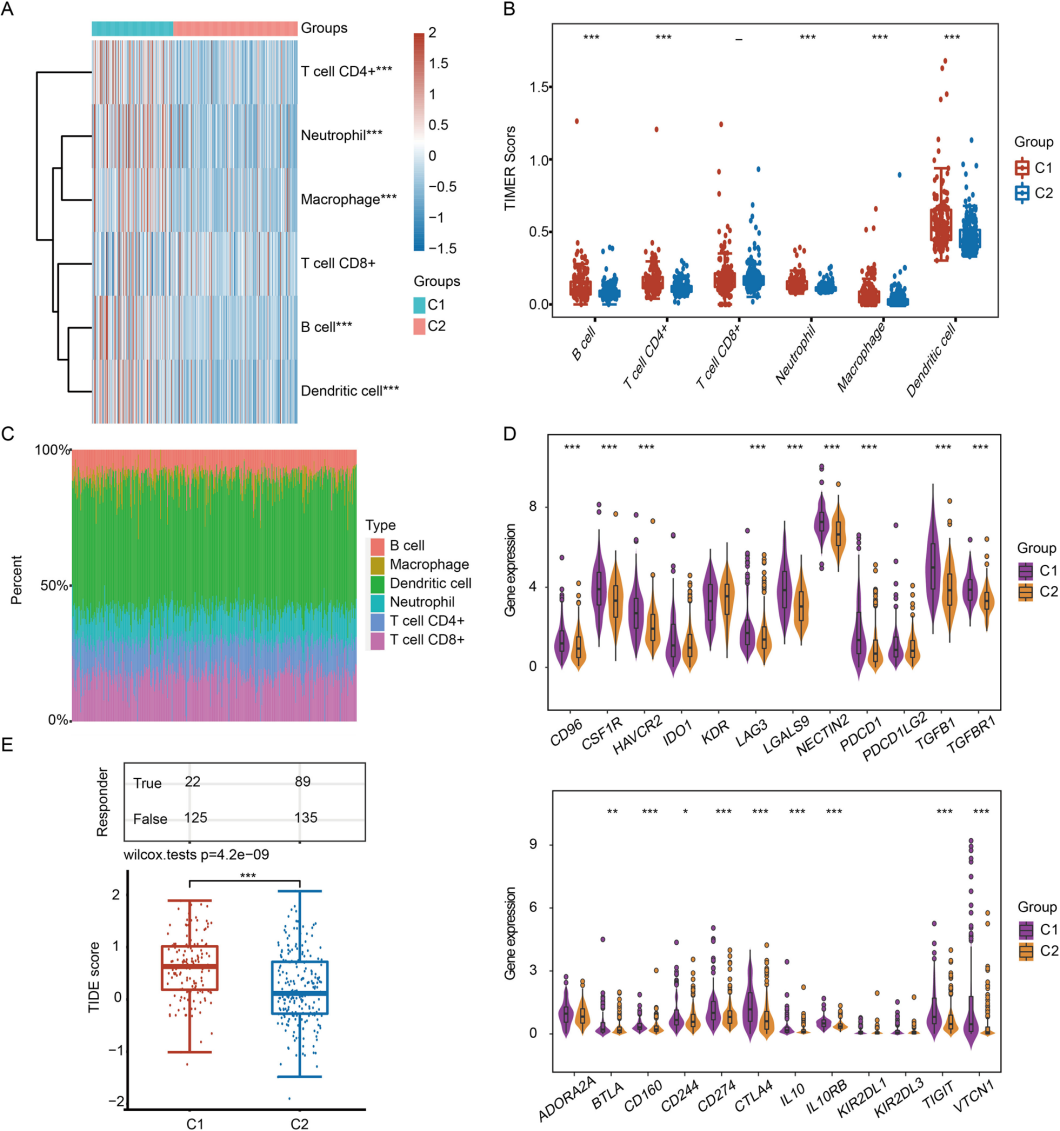
Identification of the differences in immune cell infiltration between C1 and C2 in the TIMER database. **A** Abundance heatmap of six immune cells in C1 and C2. **B** Boxplot showing the abundances of six immune cells in C1 and C2. **C** Proportion of six immune cells in each sample. Each color represents a type of immune cell. **D** Violin plot showing the expression of immunosuppressive factors in C1 and C2. **E** Boxplot showing the TIDE scores of C1 and C2. *p < 0.05, **p < 0.01, ***p < 0.001

To deeply understand the role of TFs in the immune infiltration microenvironment of HCC, we used TIMER and xCell algorithms to analyze the immune infiltration microenvironment of patients in C1 and C2. The abundance of major immune cells, except CD8 + T cells, in C1 was significantly higher than that in C2 (Fig. 4A–C). Significant differences between C1 and C2 were observed for 21 of the 35 cell types based on the xCell method (Additional file 2: Fig. S2A-C). We then analyzed the difference in the expression of 24 immunosuppressive factors between C1 and C2 and observed that 16 immunosuppressive factors exhibited upregulated expression in C1 compared with C2 (Fig. 4D). The efficacy of immunotherapy was then evaluated by the TIDE score. The TIDE score of C1 was significantly higher than that of C2, which means that patients in C2 may benefit from treatment with immune checkpoint inhibitors (Fig. 4E).

### Tumor mutation burden analysis and drug sensitivity prediction of C1 and C2

**Fig. 5:**
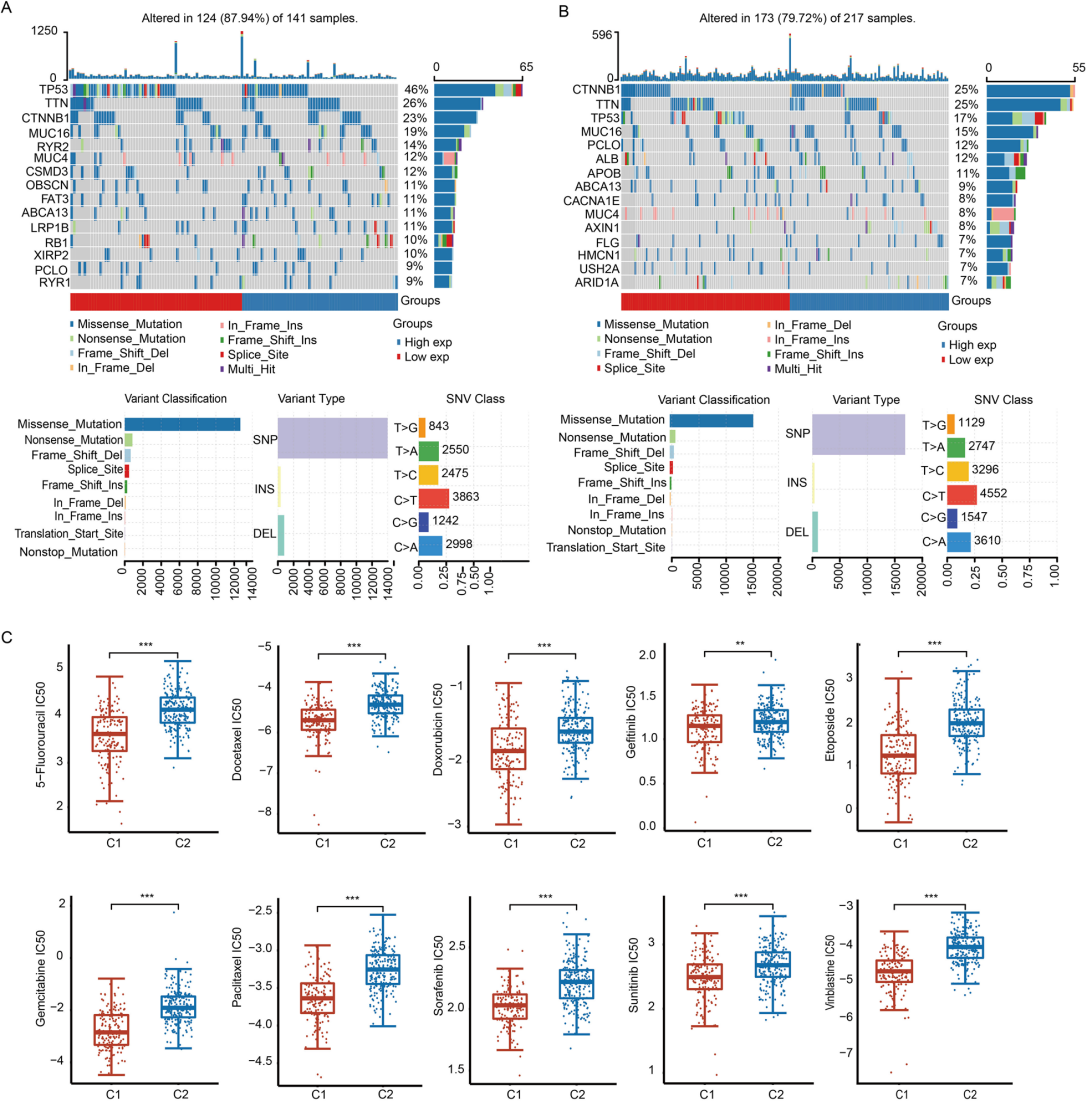
Mutational landscape and drug sensitivity in C1 and C2. **A** Waterfall plot of the mutation landscapes of C1. Mutation type and SNV classification are shown. **B** Waterfall plot of the mutation landscapes of C2. Mutation type and SNV classification are shown. **C** Boxplots of IC50 values of C1 and C2 for ten anticancer drugs according to the GDSC database. **p < 0.01, ***p < 0.001

We analyzed the difference in tumor mutational burden (TMB) between C1 and C2. The results demonstrated that the top five genes with mutations in C1 patients were *TP53*, *TTN*, *CTNNB1*, *MUC16* and *RYR2*. The top five mutated genes in C2 patients were *CTNNB1*, *TTN*, *TP53*, *MUC16* and *PCLO*. The most common mutation in C1 and C2 was missense mutation, SNP accounted for the majority, and the most common SVN class was C > T (Fig. 5A, B).

We evaluated the sensitivity of 10 anticancer drugs in C1 and C2, including 5-fluorouracil, docetaxel, doxorubicin, gefitinib, etoposide, gemcitabine, paclitaxel, sorafenib, sunitinib and vinblastine, based on the GDSC database. The IC50 value of these 10 drugs in C1 patients was significantly lower than that in C2 patients, indicating that patients in C1 were more sensitive to these anticancer agents than patients in C2 (Fig. 5C).

### Univariate Cox regression to establish a risk score prognostic model

**Fig. 6:**
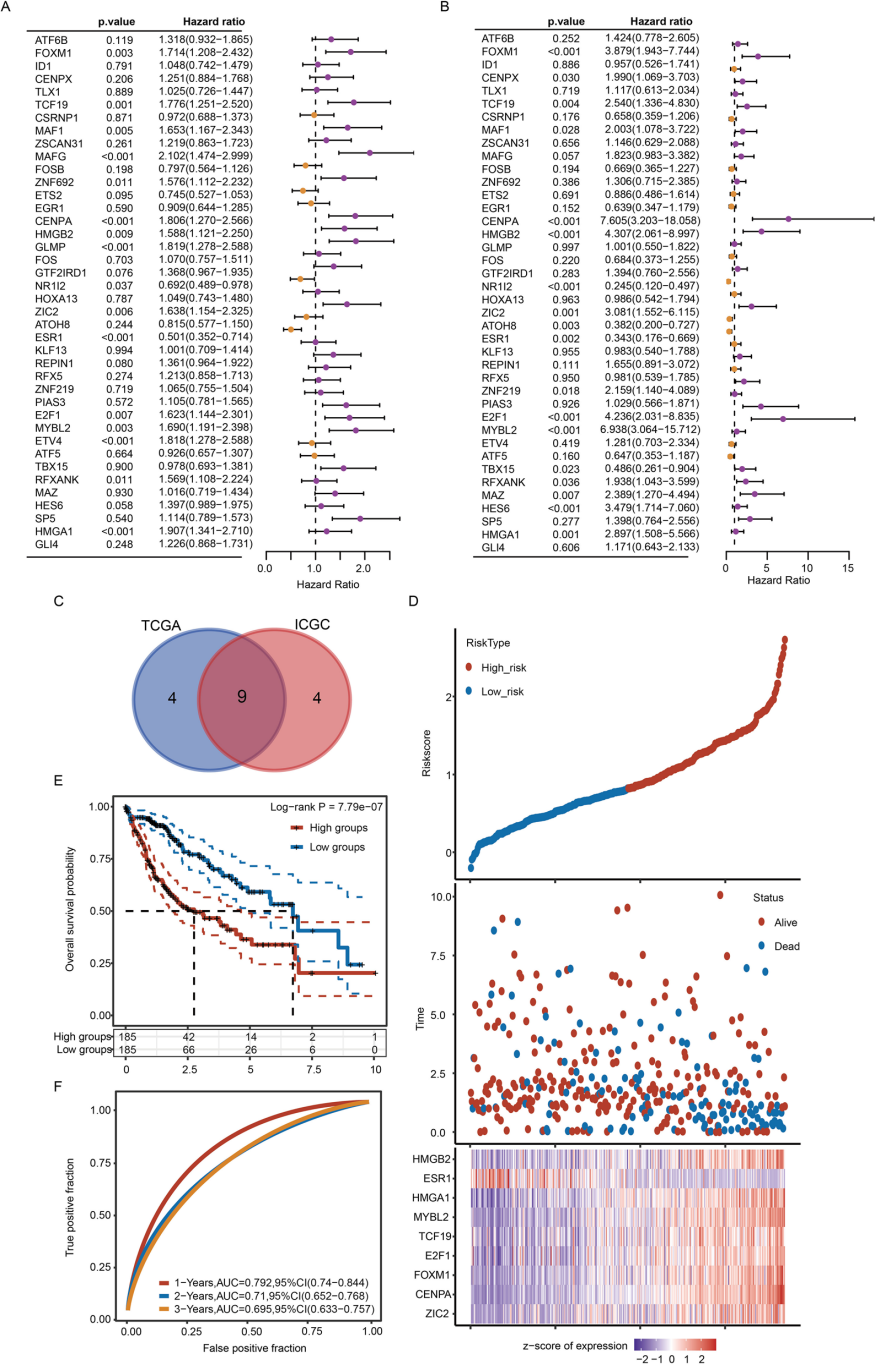
Cox regression was used to establish a risk scoring model. **A**, **B** Forest plots demonstrating the prognostic potential of 40 TFs through univariate Cox regression in the TCGA and ICGC databases. **C** Venn diagram showing the intersection of prognostic genes screened from the TCGA database and ICGC database. **D** The risk scoring model divided HCC patients into a high-risk group and a low-risk group. **E** KM analysis of OS in the high-risk group and low-risk group. **F** The AUC of time-dependent ROC curves was used to test the accuracy of model prediction

We screened TFs with potential prognostic value in the TCGA database and ICGC database through univariate Cox regression analysis (Fig. 6A, B). In total, 13 TFs were screened in the TCGA and ICGC databases with *p* < 0.05. Nine of these 13 genes overlapped (HMGB2, ESR1, HMGA1, MYBL2, TCF19, E2F1, FOXM1, CENPA and ZIC2), and we used these nine genes to establish the TF-related prognostic model (Fig. 6C). We established a risk scoring model with these 9 TFs through multivariate Cox regression in the TCGA database. The calculation formula of the risk score is Risk score = (0.084) × ZIC2 + (0.5593) × CENPA + (0.1152) × FOXM1 + (−0.0138) × E2F1 + (−0.2257) × TCF19 + (−0.0814) × MYBL2 + (0.1576) × HMGA1 + (0.0697) × ESR1 + (−0.1183) × HMGB2. According to the risk score, patients were divided into a high-risk group and a low-risk group (Fig. 6D and Additional file 9: Table S1). The survival rate of patients in the high-risk group was significantly lower than that of patients in the low-risk group (Fig. 6E). We evaluated the prognostic value of the model in 1-year, 2-year, 3-year survival rates. The area under the ROC curve (AUC) was used to evaluate the accuracy of prediction. The AUCs of the 1-year, 2-year and 3-year survival rates were 0.792, 0.71 and 0.695, respectively (Fig. 6F).

The accuracy of this TF-related prognostic model was further verified by the ICGC database. We also used multivariate Cox regression to establish a similar regression model to calculate the risk score and divided patients into a high-risk group and a low-risk group (Additional file 3: Fig. S3A and Additional file 10: Table S2). Risk score = (0.4355) × ZIC2 + (0.12) × CENPA + (0.625) × FOXM1 + (0.0677) × E2F1 + (−0.2025) × TCF19 + (0.1552) × MYBL2 + (-0.0752) × HMGA1 + -0.5791 × ESR1 + (−0.1339) × HMGB2. Similar to the results in the TCGA database, the survival rate of the high-risk group was significantly lower than that of the low-risk group (Additional file 3: Fig. S3B). Furthermore, the model was also ideal for the prognosis of patients. The AUC of the 1-year survival rate was 0.762, the 2-year survival rate was 0.807, and the 3-year survival rate was 0.826 (Additional file 3: Fig. S3C).

In addition, the risk scoring model established by nine TFs had prognostic value according to different clinicopathological parameters. In terms of age, sex, early grade (G1 + G2), advanced grade (G3 + G4), early stage (T1 + T2), advanced stage (T3 + T4), M0 and N0, TNM stage I + II and TNM stage III + IV groups, the OS of the high-risk group was significantly lower than that of the low-risk group (Additional file 4: Fig. S4).

### Cox regression and nomogram model predict the survival rate of patients

Univariate and multivariate Cox regression analyses were applied to verify the prognostic potential of the TF-related risk score in HCC patients. In univariate Cox regression, the expression of nine TFs, T-stage and M-stage were significantly correlated with the OS of HCC patients (Additional file 5: Fig. S5A). In multivariate Cox regression analysis, CENPA and T-stage were markedly associated with survival rate (Additional file 5: Fig. S5B). We then used CENPA and T-stage as variables to construct a nomogram model. The model had a good prediction effect on the 1-year, 3-year and 5-year OS of HCC patients, and the C-index value was 0.726 (Additional file 5: Fig. S5C). Calibration plots were used to visualize the prediction effect, and the predicted value was consistent with the actual value (Additional file 5: Fig. S5D).

### Analyses of expression levels and drug sensitivity of nine TFs

**Fig. 7:**
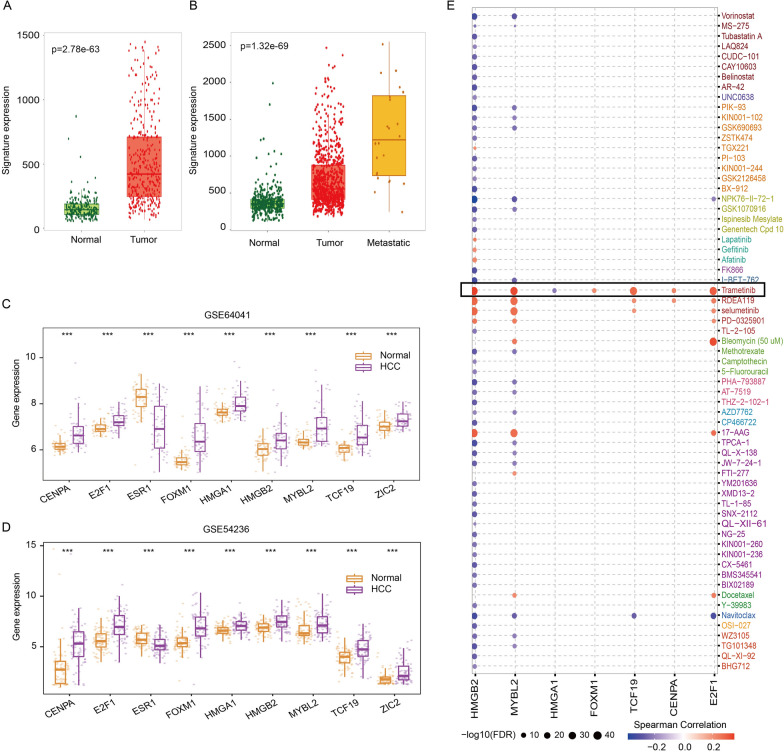
Analyses of expression levels and drug sensitivity of core TFs. **A** Gene-chip expression levels of the TF-related signature in the TNMplot database. **B** RNA-seq expression levels of the TF-related signature in the TNMplot database. **C**, **D** Expression of nine TFs in HCC samples and normal samples in the GSE64041 and GSE54236 datasets. **E** The relationship between the expression of seven TFs and chemotherapeutic drugs was explored based on the GSCALite database. ***p  < 0.001

The differential expression of nine-TF signature in normal tissues and HCC tissues was analyzed in the TNMplot database. Gene-chip and RNA-seq data showed that the expression of the signature in HCC tissues was obviously upregulated compared with normal tissues and much higher in metastatic tissues (Fig. 7A, B). The expression of nine TFs was also validated in the GEO database. The expression of CENPA, E2F1, FOXM1, HMGA1, HMGB2, MYBL2, TCF19 and ZIC2 was upregulated and ESR1 was downregulated in HCC samples compared with normal tissues based on the GSE64041 and GSE54236 datasets (Fig. 7C, D). We analyzed the relationship between the expression of TFs and drug sensitivity of HCC in the GSCALite database and found that these TFs were positively correlated with many chemotherapeutic agents. Among these drugs, trametinib was highly correlated with the expression of seven TFs. Interestingly, HCC patients with high expression of HMGB2, MYBL2, HMGA1, FOXM1, TCF19, CENPA or E2F1 exhibited higher resistance to trametinib (Fig. 7E).

**Fig. 8:**
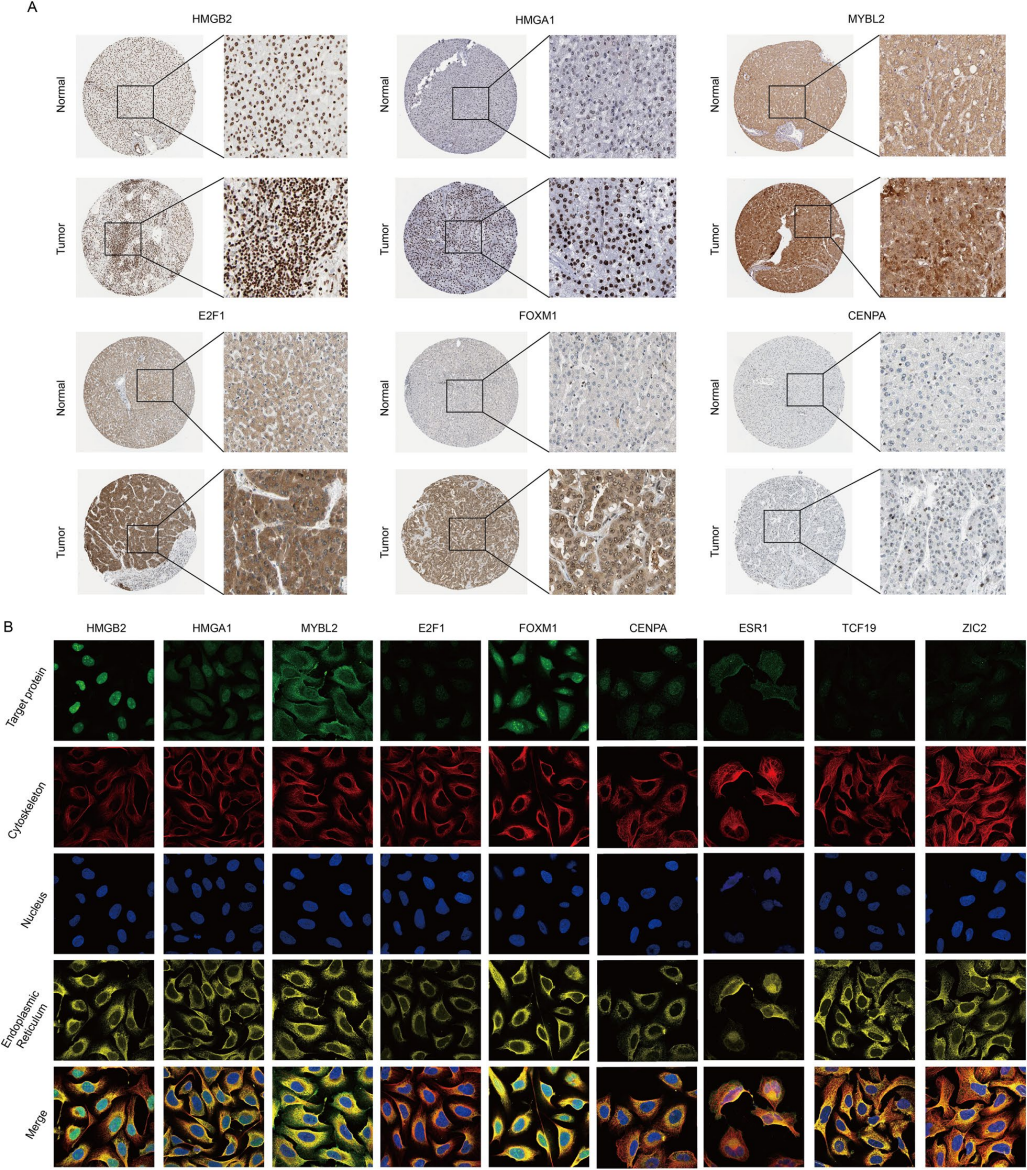
Protein expression and subcellular localization of hub TFs. **A** IHC results of TFs were obtained from the HPA database. **B** Immunofluorescence images were downloaded from the HPA database to show the cellular localization of core TFs

We further analyzed the IHC results of nine TFs in the HPA database for verification. Consistent with the results of the GEO database, the protein levels of CENPA, E2F1, FOXM1, HMGA1, HMGB2 and MYBL2 in HCC tissues were increased (Fig. 8A), and only the protein expression of ESR1 in HCC tissues was decreased (Additional file 7: Fig. S7A). We further downloaded immunofluorescence results in the HPA to carefully study the subcellular localization of nine TFs (Fig. 8B). The results showed that CENPA, E2F1, HMGA1, HMGB2, TCF19 and ZIC2 were mainly localized in the nucleus. FOXM1 and MYBL2 were detected in both the nucleus and cytoplasm. ESR1 is mainly localized on vesicles. We also collected single-cell RNA-seq data of nine TFs from the Human Life Browser database (Additional file 6: Fig. S6). Nine TFs were expressed in HCC cells and many other types of liver cells. In addition, the expression of nine TFs was also detected in immune cells (such as T cells and B cells) (Additional file 6: Fig. S6).

### Binding site prediction of trametinib with nine TFs

**Fig. 9:**
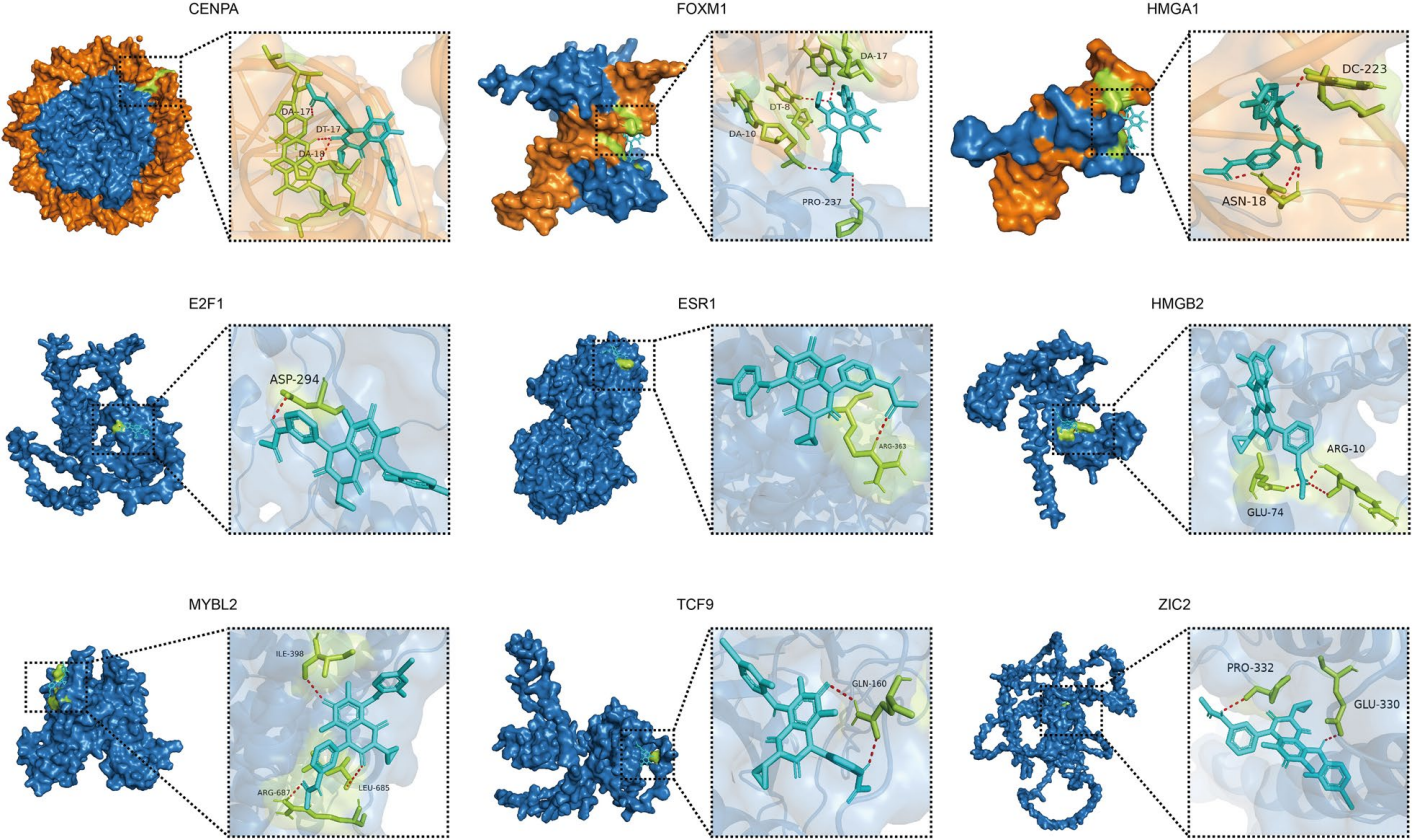
Molecular docking analyses. Predicted binding sites between trametinib and nine TFs. Orange represents DNA, blue represents protein, light blue represents Trametinib, and yellow represents the residue of drug docking. Red dotted lines represent possible hydrogen bonds

We performed molecular docking of nine TFs and trametinib (Fig. 9 and Table 3). In the predicted results, trametinib interacts with DNA bound by CENPA. Trametinib binds to PRO-237 of FOXM1 and binds to DNA near the DNA binding site of FOXM1. Trametinib binds to ASN-18 of HMGA1 and DNA. Trametinib binds to E2F1 by interacting with the amino acid ASP-294. Trametinib binds to ARG-363 of ESR1. Trametinib binds to ARG-10 and GLU-74 of HMGB2. Trametinib binds to ILE-398, LEU-685 and ARG-687 of MYBL2. Trametinib binds to GLN-160 of TCF19. Trametinib binds to PRO-332 and GLU-330 of ZIC2. More importantly, the docking energy between trametinib and the core 8 genes was less than –5 kcal/mol (Table 3).

**Table 3:** Predictive binding energy of core proteins to trametinib

Gene symbol	Drug	Predictive binding energy (Kcal/mol)
HMGB2	Trametinib	−6.19
HMGA1	Trametinib	−7.31
FOXM1	Trametinib	−7.95
TCF19	Trametinib	−6.82
MYBL2	Trametinib	−6.27
CENPA	Trametinib	−6.77
ESR1	Trametinib	−5.92
E2F1	Trametinib	−4.48
ZIC2	Trametinib	−5.28

### Correlation analysis between the expression levels of nine TFs and trametinib sensitivity

**Fig. 10:**
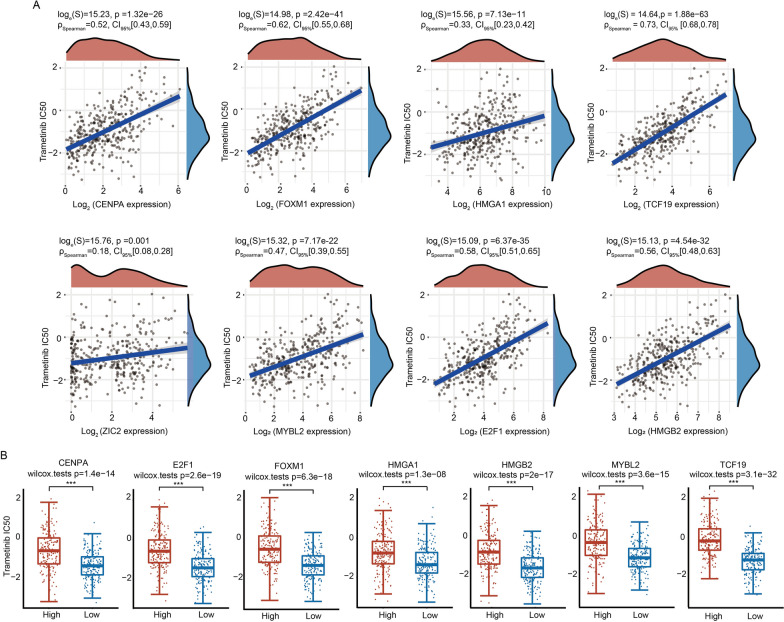
Drug sensitivity analysis of nine TFs with trametinib. **A** Correlation between the expression levels of eight TFs and trametinib drug sensitivity. **B** The effects of the expression of core TFs on the IC50 value of trametinib. ***p < 0.001

To further explore the association of trametinib with the nine TFs, we analyzed the correlations between the expression levels of nine TFs and the IC50 values of trametinib in the GDSC database. The results indicated that the expression levels of CENPA, FOXM1, HMGA1, TCF19, ZIC2, MYBL2, E2F1 and HMGB2 were positively correlated with the IC50 values of trametinib (Fig. 10A). However, no correlation was observed between ESR1 expression and trametinib sensitivity (*p* > 0.05) (Additional file 7: Fig. S7B). Moreover, HCC patients were divided into a high-expression group and a low-expression group according to the expression levels of nine TFs, respectively. Drug sensitivity results directly indicated that HCC cells with high expression of CENPA, E2F1, FOXM1, HMGA1, HMGB2, MYBL2 and TCF19 were less sensitive to trametinib (Fig. 10B). There were no associations between trametinib sensitivity and ESR1 and ZIC2 in HCC (Additional file 7: Fig. S7C).

### Trametinib decreases the expression of core TFs in HCC cells

**Fig. 11:**
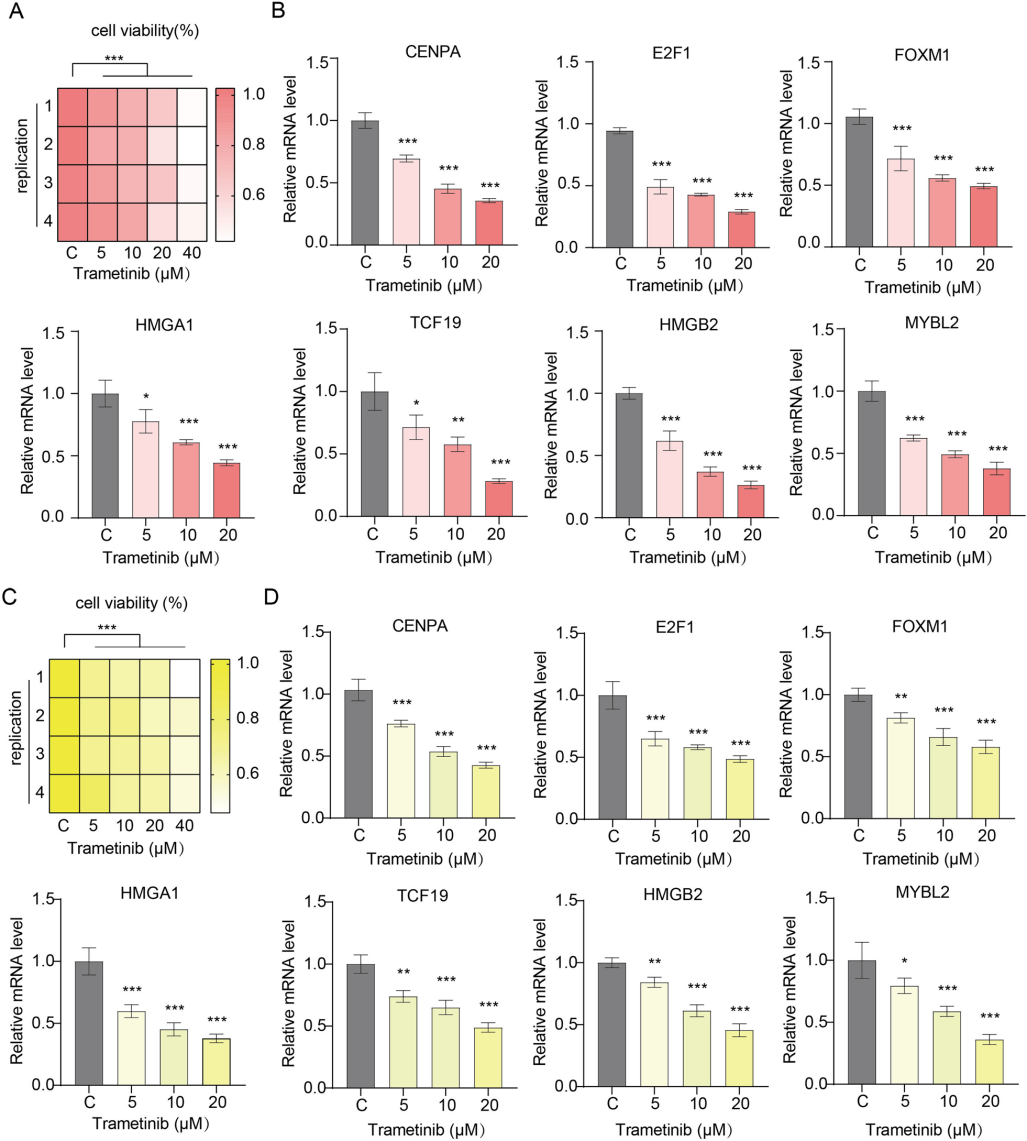
Changes in the expression levels of core TFs under trametinib treatment. **A**, **C** Cell survival rates were examined by the CCK-8 assay. HepG2 (**A**) and Huh7 (**C**) cells were treated with different concentrations of trametinib. After 24 h, cell viability was examined using the CCK-8 assay. **B**, **D** Expression of core TFs in HCC cells was examined by RT-PCR. HepG2 (**B**) and Huh7 (**D**) cells were treated with different concentrations of trametinib for 24 h. Total RNA was extracted from trametinib-treated cells. *p < 0.05, **p < 0.01, ***p < 0.001

The cell viability of HepG2 and Huh7 cells under trametinib treatment was reduced in a concentration-dependent manner (Fig. 11A, C). The expression of CENPA, E2F1, FOXM1, HMGA1, TCF19, HMGB2 and MYBL2 was significantly decreased with increasing concentrations of trametinib (Fig. 11B, D). These results suggest that trametinib may achieve its anticancer effect by downregulating the expression of TFs in HCC.

## Discussion

Carcinogenesis is a multistep process and requires constitutive expression and/or activation of TFs. How to apply TF to the prognosis evaluation of HCC patients and target TF to develop tumor drugs are significant clinical challenges. In this study, we used a variety of bioinformatics technologies to explore TFs-related clinical prognostic markers and drug targets in HCC. We employed consensus clustering analysis to classify patients into two distinct molecular subtypes (C1 and C2) based on the expression pattern of TFs. The clinical prognosis of C1 patients was poorer than that of C2 patients (Fig. 3). Most TFs were highly expressed in C1 and defined as oncogenes, such as HMGA1, ETV4 and MAZ (Additional file 1: Fig. S1) [18]. In drug sensitivity prediction, C1 patients were more sensitive to several common chemotherapy drugs compared with C2 patients (Fig. 5), indicating that HCC patients in the C1 subgroup are more suitable for chemotherapeutics. Therefore, this TFs-based classification and the construction of prognosis model can help judge the prognosis of HCC patients and implement personalized treatment from the perspectives of predictive, preventive and personalized medicine.

Although the differences in the prognosis and efficacy of chemotherapy between C1 and C2 were evaluated, we noticed that not all 40 differentially expressed TFs were correlated with the prognosis of HCC patients (Fig. 6A, B). Moreover, due to the large number of genes, it is difficult to apply them to the clinical diagnosis of HCC patients. Considering the possibility of future clinical applications, we screened 9 prognostic core TFs from 40 genes using Cox regression analysis (Fig. 6D, E). The risk score was further calculated according to the expression level of 9 core TFs, and HCC patients were separated into a high-risk group and a low-risk group. The prognosis of the high-risk group was poorer than that of the low-risk group (Fig. 6E). In addition, the prognostic potential of this model was confirmed by the AUC values (Fig. 6F). Thus, this prognostic risk score model showed good predictive and discerning ability in HCC patients. Among these nine genes, HMGB2, HMGA1, MYBL2, TCF19, E2F1, FOXM1, CENPA and ZIC2 were obviously upregulated in HCC samples. In contrast, ESR1 was significantly downregulated in HCC samples (Fig. 8). ESR1 was expressed at lower levels in HCC tissues than in normal tissues and correlated with poorer prognosis in HCC patients [19, 20]. The downregulation of ESR1 may be related to the carcinogenic driving force of HCC.

More importantly, we also identified trimetinib as a potential drug targeting core dysregulated TFs in the prediction of drug sensitivity. The expression levels of seven TFs were positively correlated with the IC50 value of trametinib in HCC cells (Fig. 10). Through molecular docking analysis, trametinib had good binding energy with key TFs, indicating that trametinib may be a potential drug targeting TFs in HCC patients (Fig. 9). Notably, the predicted binding sites of trametinib and CENPA, FOXM1 and HMGA1 are near the binding sites of DNA and protein, and the drug is predicted to interact with DNA. These results suggest that trametinib may affect the binding process of TFs and DNA. When HCC cells were treated with trametinib, the expression of seven TFs was downregulated in a dose-dependent manner (Fig. 11). However, drug sensitivity analysis indicated that HCC patients with high expression of key TFs were more resistant to trametinib, so the decreased expression of TFs might be responsible for the anticancer effect of trametinib.

Previous studies have identified TF-related gene signatures and risk score models in colorectal cancer, glioblastoma, breast cancer, endometrial cancer, ovarian cancer and gastric cancer, suggesting that TF-based signatures can predict prognosis and therapeutic efficacy for cancer patients [7–14]. In addition, a 2-TF prognostic signature with different clinical features and treatment preferences was constructed in HCC [15]. In the present study, we constructed a 9-TF prognostic signature and explored its potential to guide clinical treatment using completely different analytical strategies. The AUC values of the 2-TF prognostic signature were 0.59–0.74 for 1-, 2- and 3-year OS in different databases according to Yang’s study [15]. In contrast, the AUC values of the 9-TF prognostic signature in our study were 0.695–0.826 for 1-, 2- and 3-year OS, indicating that the 9-TF prognostic signature we established may have better performance in predicting OS in HCC patients. Moreover, decision curve analysis (DCA) is increasingly used in research for diagnostic testing and/or evaluation of predictive models. We also performed a DCA to compare the differences between these two TF-based models. In fact, compared with the previously estimated 2-TF prognostic signature, this 9-TF prognostic signature we established is more sensitive and effective in predicting OS in HCC patients (Additional file 7: Fig. S7D). However, external validation in the clinic is also important for evaluating the accuracy of the predictive power of the gene signature in an independent patient cohort.

Additionally, Yang et al. did not investigate the relationship between TF-based signature and immune cell infiltration in HCC [15]. Here, we explored the potential associations between TFs and immune infiltration and immunotherapy. In terms of immune infiltration, we used the TIMER algorithm to calculate the infiltration abundance of six immune cells. The infiltrated abundance of major immune cells in C1 was higher than that in C2 (Fig. 4), indicating that patients with different subtypes had differences in immune cell infiltration, which may cause different responses to chemotherapy and immunotherapy. Consistently, the expression of most immunosuppressive factors in C1 was significantly higher than that in C2 (Fig. 4D). These phenomena suggest that the dysregulation of the TF-mediated transcriptional regulatory network may affect the state of the tumor immune microenvironment. The TIDE score indicated that C1 patients were more prone to immune escape during immunotherapy (Fig. 4E). Our analysis results revealed that HCC patients with different TF-related molecular subtypes were suitable for different treatments, which provided great help for precise medical treatment of HCC.

In summary, we identified two TF-related molecular subtypes in HCC patients. This cluster can be applied to survival prediction, individualized chemotherapy, and immunotherapeutic guidance for patients with HCC. The risk score model composed of nine TFs-related signatures performed well in HCC patient risk prediction. In the drug sensitivity analysis, the potential interaction sites of nine TFs with trametinib were predicted by molecular docking. Trametinib exhibited anticancer effect probably by reducing the expression levels of core oncogenic TFs. The precise molecular mechanism by which trimetinib inhibits the expression of these core TFs and the signaling pathways and downstream targets regulated by trimetinib through TFs will be the focus of our subsequent studies. Additionally, we also hope to screen novel new inhibitors that simultaneously target multiple dysfunctional TFs to improve the therapeutic effect of HCC. Our findings help researchers to deeply understand the pathological molecular mechanisms of HCC at the transcriptional regulation level, identify TF-related prognostic biomarkers for HCC, and provide important clues for exploring TFs as drug targets in HCC.

### Supplementary Information

**Additional file 1: Figure S1.** Analysis of DEGs in the ICGC database and the expression of 40 TFs in C1 and C2. (A) Volcano plot of DEGs between HCC tissues and normal tissues. |Log_2_(Fold Change)|> 1 and p < 0.05 were defined as thresholds. (B) Clustering heatmap of the expression of DEGs in HCC samples and normal samples. (C) Expression of 40 TFs in C1 and C2. *p < 0.05, **p < 0.01, ***p < 0.001.

**Additional file 2: Figure S2.** Immune cell infiltration between C1 and C2 according to the xCell database. (A) Heatmap showing different infiltrated abundances of immune cells. (B) Boxplot and scatter plot demonstrating the differences in the infiltrated abundance of immune cells in C1 and C2. (C) Proportion of immune cell composition in each sample. *p < 0.05, **p < 0.01, ***p < 0.001.

**Additional file 3: Figure S3.** Risk scoring model based on the ICGC database. (A) HCC patients were divided into a high-risk group and a low-risk group according to the risk score. (B) KM analysis of OS in the high-risk and low-risk groups. (C) The AUC of time-dependent ROC curves was generated to test the accuracy of model prediction.

**Additional file 4: Figure S4.** Prognostic value of the risk scoring model in terms of different clinical parameters. KM analysis of OS in high-risk and low-risk groups in terms of different clinicopathological classifications.

**Additional file 5: Figure S5.** Construction of a nomogram integrating predictive factors to verify the prognostic ability. (A, B) Univariate and multivariate Cox regression analyses were used to identify the independent prognostic factors from TFs and various clinical parameters. (C) A nomogram integrating prognostic characteristic variables was constructed to predict the 1-year, 2-year, and 3-year OS of HCC patients. (D) Calibration curves were generated to check the reliability of the predicted and actual values.

**Additional file 6: Figure S6.** Single-cell RNA-seq analysis of nine TFs using the Human Life Browser database. Nine TFs were expressed in different types of liver cells and immune cells.

**Additional file 7: Figure S7.** The expression of TFs and drug sensitivity. (A) IHC results of ESR1 in HPA database. (B) Correlation between ESR1 expression and IC50 values of trametinib. (C) Differences in IC50 values of trametinib between high and low expression groups of ESR1 and ZIC2. (D) Decision curve analysis (DCA) of 9-TF prognosis signature and previous 2-TF prognosis signature.

**Additional file 8: Table S1.** The raw data for calculating the risk score based on the TCGA database.

**Additional file 9: Table S2.** The raw data for calculating the risk score based on the ICGC database.

## Data Availability

The datasets generated and analyzed during the current study are available in the TCGA (https://portal.gdc.cancer.gov), ICGC (https://dcc.icgc.org/releases/current/Projects) and GEO (https://www.ncbi.nlm.nih.gov/geo/) databases.
