# Supplementary figures and images for: Transcription factors-related molecular subtypes and risk prognostic model: exploring the immunogenicity landscape and potential drug targets in hepatocellular carcinoma
Source: Cancer Cell Int. 2024 Jan 4;24:9. doi: 10.1186/s12935-023-03185-1 (PMC10765642; doi:10.1186/s12935-023-03185-1)

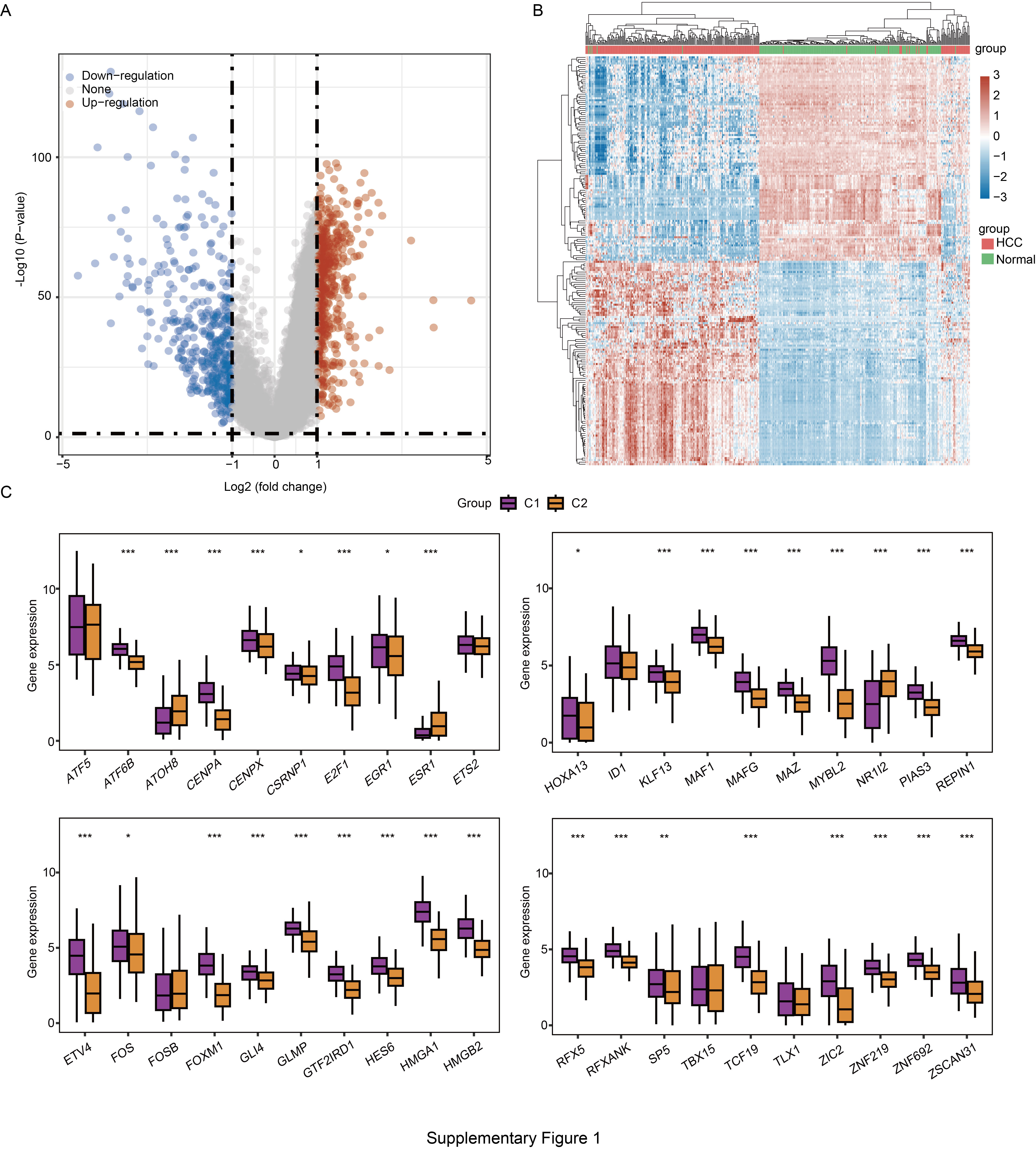

Supplement: Supplementary file 1 — Additional file 1: Figure S1. Analysis of DEGs in the ICGC database and the expression of 40 TFs in C1 and C2. (A) Volcano plot of DEGs between HCC tissues and normal tissues. |Log2(Fold Change)|> 1 and p < 0.05 were defined as thresholds. (B) Clustering heatmap of the expression of DEGs in HCC samples and normal samples. (C) Expression of 40 TFs in C1 and C2. *p < 0.05, **p < 0.01, ***p < 0.001. [file 12935_2023_3185_MOESM1_ESM.jpg]

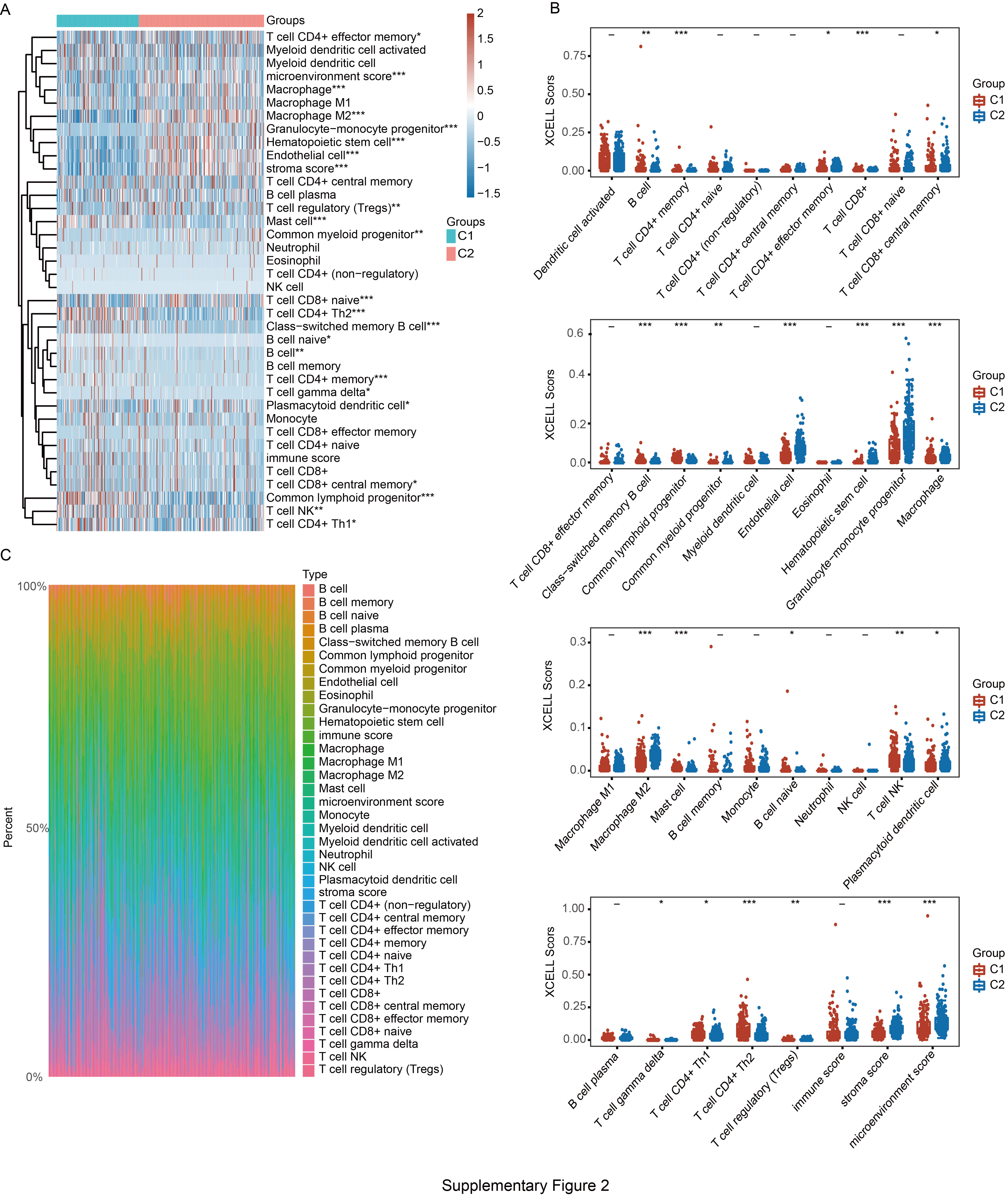

Supplement: Supplementary file 2 — Additional file 2: Figure S2. Immune cell infiltration between C1 and C2 according to the xCell database. (A) Heatmap showing different infiltrated abundances of immune cells. (B) Boxplot and scatter plot demonstrating the differences in the infiltrated abundance of immune cells in C1 and C2. (C) Proportion of immune cell composition in each sample. *p < 0.05, **p < 0.01, ***p < 0.001. [file 12935_2023_3185_MOESM2_ESM.jpg]

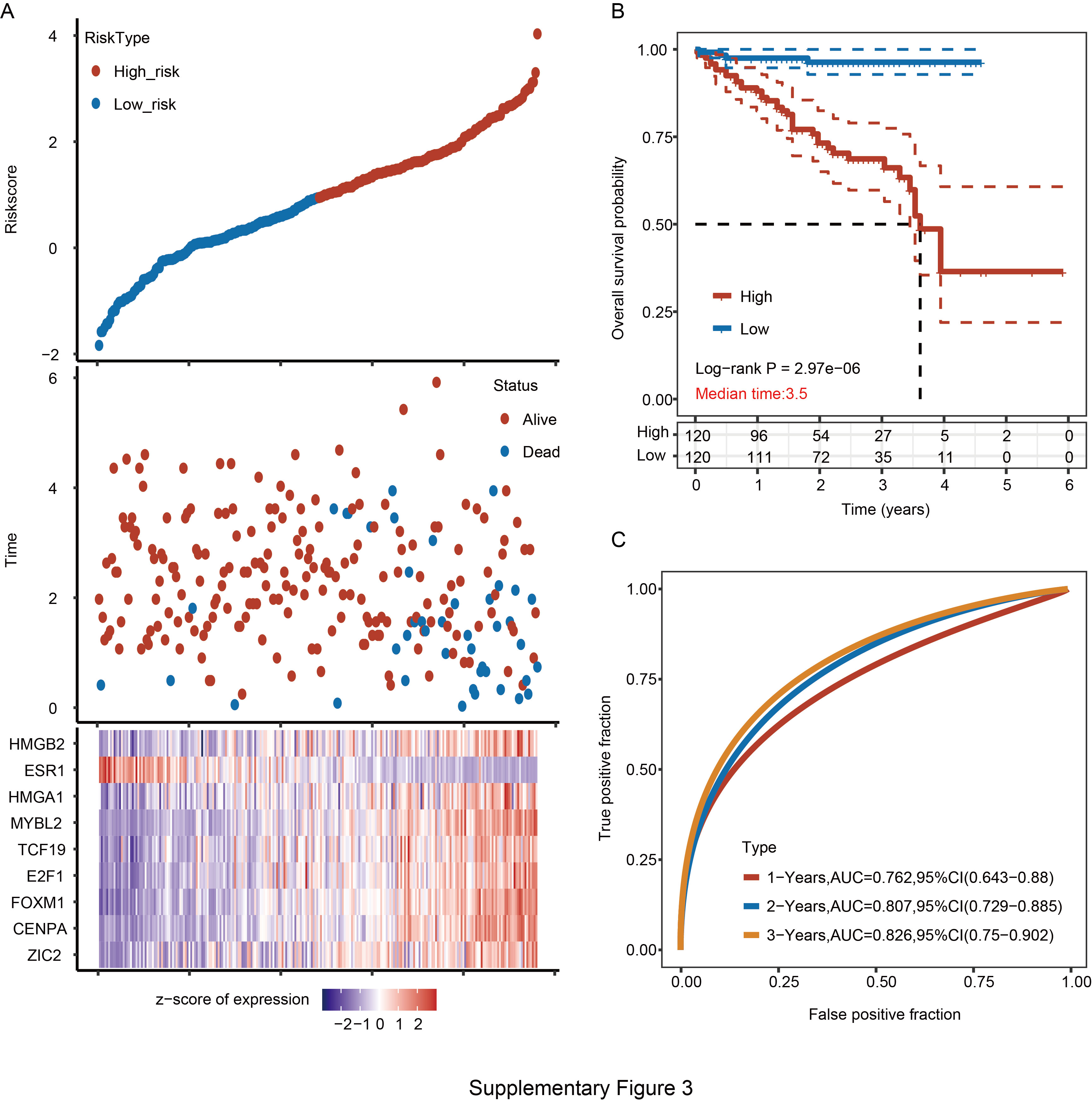

Supplement: Supplementary file 3 — Additional file 3: Figure S3. Risk scoring model based on the ICGC database. (A) HCC patients were divided into a high-risk group and a low-risk group according to the risk score. (B) KM analysis of OS in the high-risk and low-risk groups. (C) The AUC of time-dependent ROC curves was generated to test the accuracy of model prediction. [file 12935_2023_3185_MOESM3_ESM.jpg]

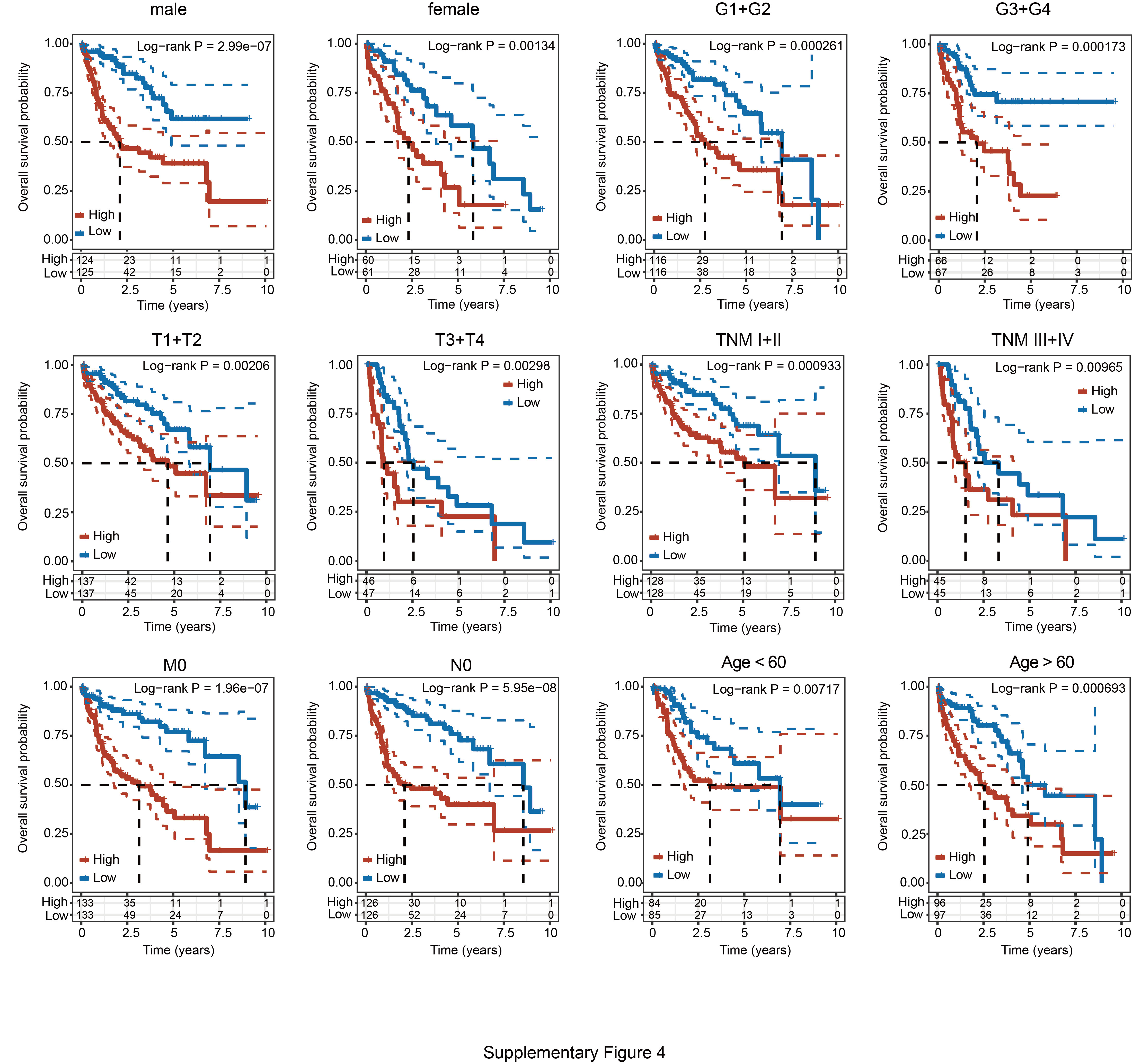

Supplement: Supplementary file 4 — Additional file 4: Figure S4. Prognostic value of the risk scoring model in terms of different clinical parameters. KM analysis of OS in high-risk and low-risk groups in terms of different clinicopathological classifications. [file 12935_2023_3185_MOESM4_ESM.jpg]

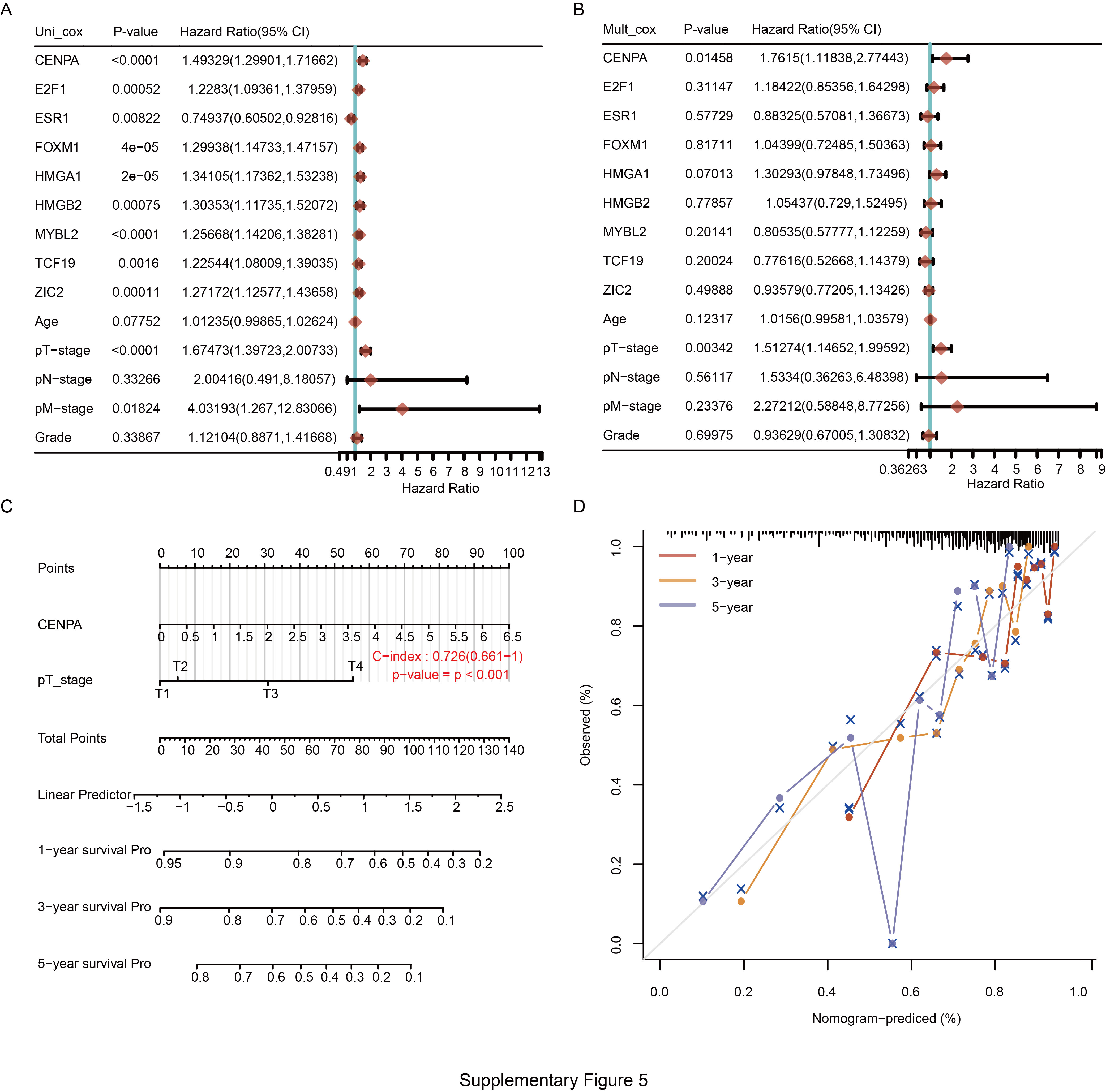

Supplement: Supplementary file 5 — Additional file 5: Figure S5. Construction of a nomogram integrating predictive factors to verify the prognostic ability. (A, B) Univariate and multivariate Cox regression analyses were used to identify the independent prognostic factors from TFs and various clinical parameters. (C) A nomogram integrating prognostic characteristic variables was constructed to predict the 1-year, 2-year, and 3-year OS of HCC patients. (D) Calibration curves were generated to check the reliability of the predicted and actual values. [file 12935_2023_3185_MOESM5_ESM.jpg]

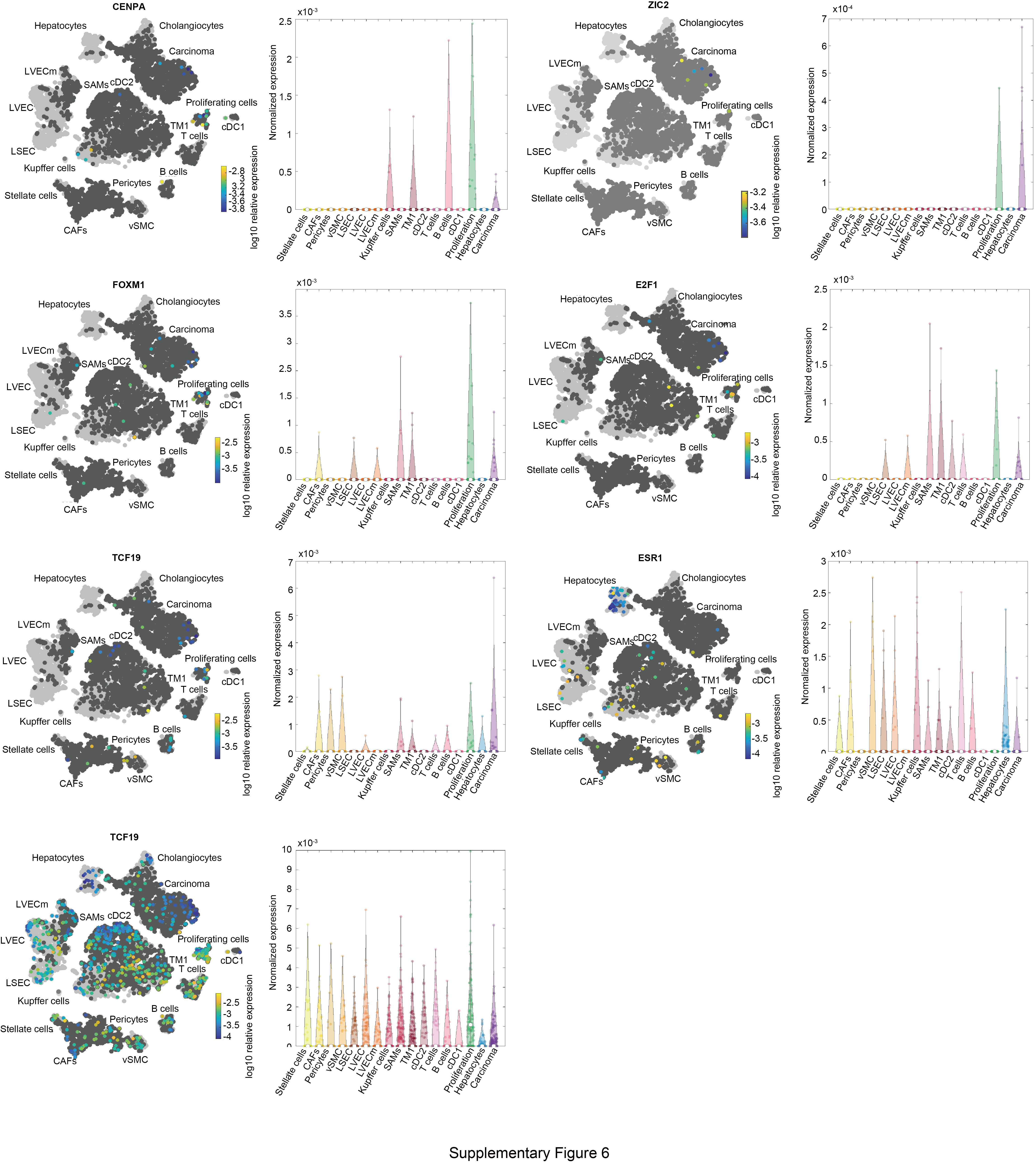

Supplement: Supplementary file 6 — Additional file 6: Figure S6. Single-cell RNA-seq analysis of nine TFs using the Human Life Browser database. Nine TFs were expressed in different types of liver cells and immune cells. [file 12935_2023_3185_MOESM6_ESM.jpg]

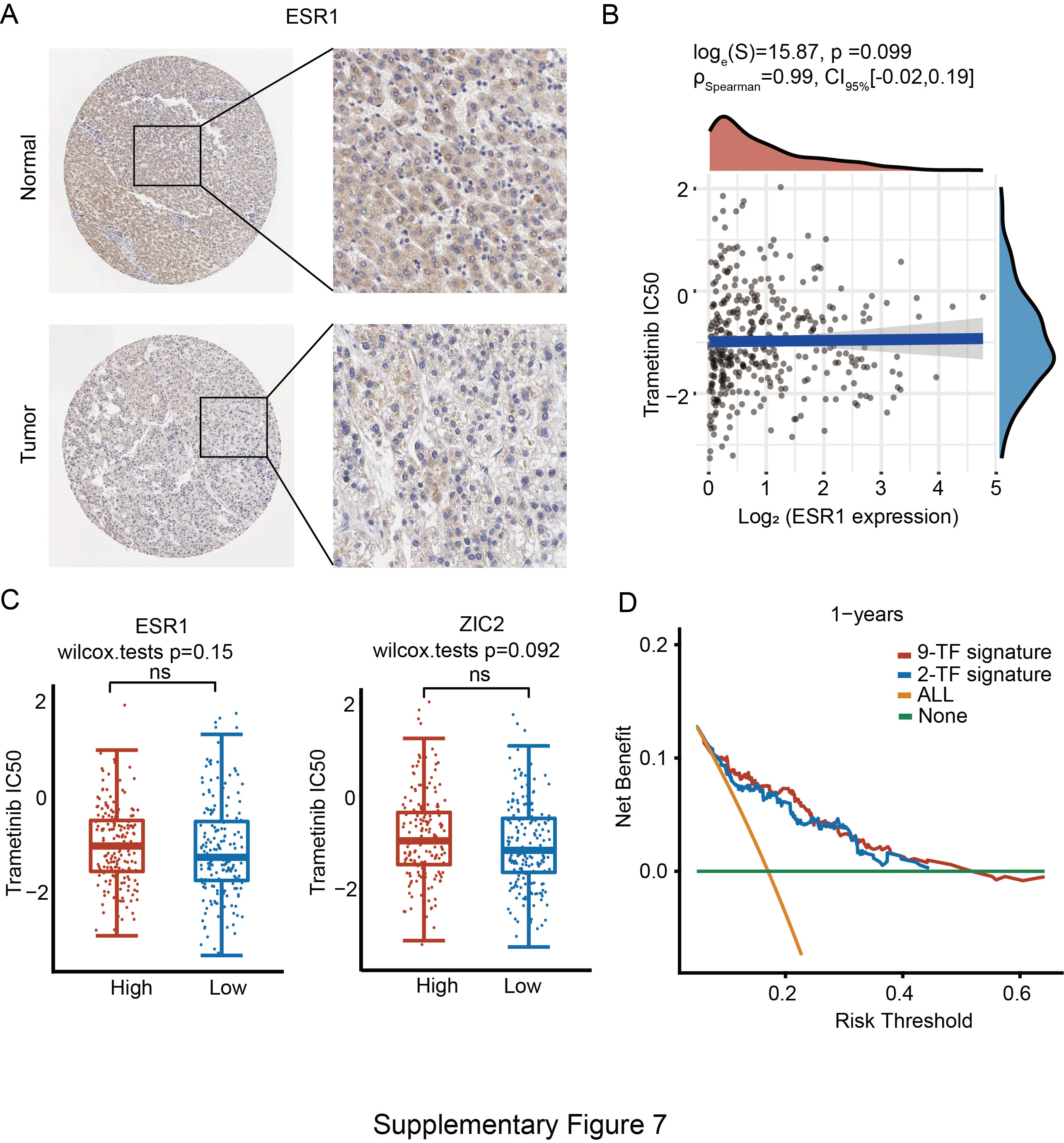

Supplement: Supplementary file 7 — Additional file 7: Figure S7. The expression of TFs and drug sensitivity. (A) IHC results of ESR1 in HPA database. (B) Correlation between ESR1 expression and IC50 values of trametinib. (C) Differences in IC50 values of trametinib between high and low expression groups of ESR1 and ZIC2. (D) Decision curve analysis (DCA) of 9-TF prognosis signature and previous 2-TF prognosis signature. [file 12935_2023_3185_MOESM7_ESM.jpg]
